# Continental diatom biodiversity discovery and description in China: 1848 through 2019

**DOI:** 10.3897/phytokeys.160.54193

**Published:** 2020-09-08

**Authors:** J. Patrick Kociolek, Qingmin You, Qi Liu, Yan Liu, Quanxi Wang

**Affiliations:** 1 Museum of Natural History and Department of Ecology and Evolutionary Biology, University of Colorado, Boulder, USA University of Colorado Boulder United States of America; 2 College of Life Sciences, Shanghai Normal University, Shanghai 200234, P. R. China; 3School of Life Science, Shanxi University, Taiyuan, 030006, China Shanghai Normal University Shanghai China; 3 School of Life Science, Shanxi University, Taiyuan, 030006, China Shanxi University Taiyuan China; 4 College of Life Science and Technology, Harbin Normal University, Harbin, China Harbin Normal University Harbin China

**Keywords:** new taxa, diatoms, Bacillariophyta, Skvortzov, China, continental

## Abstract

In this paper we inventory the continental diatom taxa described from inland waters in China, from the first species descriptions dating back to 1848 through 2019. China’s geography and hydrography are complex, including the world’s highest mountains, many large rivers, salty lakes, and large karst regions. From this area, a total of 1128 taxa have been described from China over this time period. We examine the number of taxa described in ca. 20-year intervals and note the periods of time of no to few descriptions, versus time intervals with many taxon descriptions. Early on, taxon descriptions of freshwater diatoms from China were done by mostly by Europeans working alone, and the time frame of 1948 to 1967 had few descriptions, as a devasting famine and the cultural revolution impacted scientific work and productivity. B.V. Skvortzov produced a large number of taxon descriptions, during his time in residence in Harbin, later while in Sao Paulo, Brazil, and even posthumously. More recently, a wide range of labs and collaborations across China, and with a diverse array of international partners, is ushering in a new, robust era of research on the biodiversity of continental diatoms. A few areas of research and work for the future are discussed.

## Introduction

Asia has received considerable attention in the context of biodiversity discovery, biogeography and resolving the evolutionary history of a variety of lineages (Gower et al. 2012). It is an area harboring many endemic species and broader lineages (López-Pujol et al. 2011; Lu et al. 2018), relicts (Wu et al. 2007; López-Pujol and Ren 2009; Li et al. 2012a) as well as extinct taxa (Fu et al. 2019; Proust et al. 2020; Zhang 2020). Many diverse lineages have originated, radiated and gone extinct in China. This is true for many groups of organisms, including continental diatoms (see Skvortzov 1937; Hustedt 1938a, b, 1939; Williams 2004; Williams and Reid 2006; Kulikovskiy et al. 2012, 2015; Hamsher et al. 2014; Kociolek 2019).

Within China, there has been a long history and much recent attention on the description of many new species and even genera from continental ecosystems across the country. Interest in continental diatoms of China extends beyond biodiversity discovery to a rich array of work related to water quality and bioassessment (e.g. Ouyang et al. 2015), impacts of eutrophication and the creation of dams (Wang and Zhang 2004; Shen et al. 2018), paleoenvironmental reconstructions (Rioual and Wang 2009) and the development of many products with diatoms (Zhang et al. 2012; Wang and Seibert 2017; Zhang 2019).

The work on biodiversity discovery, as well as ecological work and more applied studies, depends on a working knowledge of the flora that has already been documented. We have compiled and present here a listing of the continental diatoms described from China to provide these descriptive and practical projects with a historical context and a baseline against future work can be compared. This compilation of new taxa described from China, and the publications in which they were presented, can also help interpret the history and development of diatom studies in China, from the middle of the 19^th^ century to the present.

## Methods

In our work developing this compilation of names of the continental diatoms described from China, we used the current geo-political circumscription of the country recognized by the United Nations. Our definition of “continental” refers to a variety of inland waters bodies, including freshwaters as well as those with high conductivity and, to some extent, ‘salty’ waters. But we have excluded taxa described from estuaries and marine localities from our review.

The bases of this compilation are the major resources for diatom nomenclature, including Catalogue of Diatom Names (Fourtanier and Kociolek 2011), DiatomBase (Kociolek et al. 2020) and AlgaeBase (Guiry and Guiry 2020). In addition, we reviewed several of the compilations of diatoms of China (“*Flora Algarum Sinicarum Aquae Dulcis*”) and some primary literature that escaped the notice of these comprehensive works and summative projects. An important reference for this work is Jin (1951), in which the knowledge of diatoms reported from China from 1848 to 1946, noting over 1000 taxa had been reported from marine and freshwater ecosystems, is summarized. The paper lists the taxa described from China (mostly by Skvortzov up to 1946). Although Jin (1951) did not document most of the other descriptions by European authors, both prior to and concurrent with Skvortzov, and his list obviously does not include post-1946 names, it is a great (but under cited) reference from which to develop a list of diatoms from China. The Skvortzov names were checked against the check list of his taxa compiled by Gololobova (2012). All of the names documented in this work have been included in DiatomBase.

## Results

### Continental diatoms described from China: An overview

**Figure 1.**
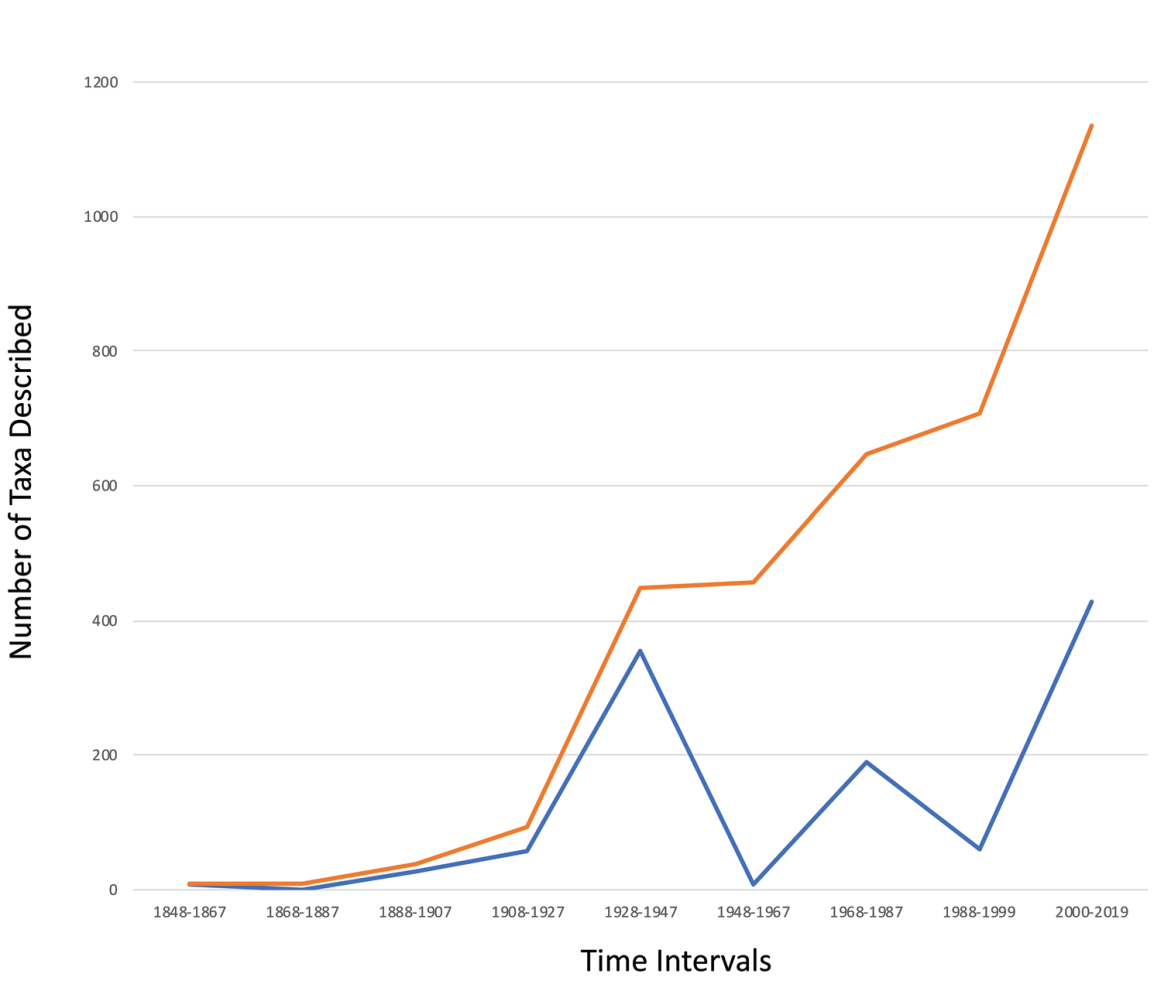
Number of species, varieties and forms described from freshwaters China, 1848–2019. The blue line represents the number of taxa described for each time interval. The orange line represents the cumulative number of taxa described over the entire period.

In the 170-year history of continental diatom discovery in China, 1128 taxa have been described at the level of species and below (Table 1). This was not a smooth, equal accumulation of species over time, and if we examine the overall time period in groups of 20-year intervals, we can see there were times when significant numbers of taxa were described. For example, the time interval of 1928 to 1947 there were 355 taxa described in 16 publications, and between 1968 and 1987, 189 taxa were described in only 13 publications. In both instances, most of the publications were by a single author (See Appendices 1 and 2). On the other hand, in the more recent period of 2000 to 2019, the highest number of taxa were described (421), and published in 99 separate publications. Many of these papers were multi-authored. Periods of low publication of new species can be found in the earliest periods (1848–1887) and in the period 1948–1967 (Fig. 1).

In the next sections, we break down the work of continental diatom discovery in China into two periods, the initial period (1848–1999) and more recent period of continental diatom discovery (2000–2019). We examine the changes in approach and productivity during these time periods and compile a list of the taxa described and the references in which they were published.

### The initial period: 1848–1999

Studies on the continental diatoms of China date back to the mid 1800’s, to the work of Ehrenberg. From these initial works through ¾ of the 20^th^ century, there were many studies that documented continental diatom taxa in China, with a few genera and many species and subspecific taxa being proposed. The majority of taxa described was at the subspecific level. In Appendix 1 we document the new genera, species and subspecific continental diatoms described from China in this time period. This list, based on more than 50 publications only, shows that there were 2 genera described from continentals of China (*Amphiraphia*Chen and Zhu 1983 and *Porosularia*Skvortzov 1976b). Neither of these genera have been reported since they were first described, and neither of these names are in use today.

Table 1 and Appendix 1 show that from 1848 until 1999, a total of 707 taxa were described from continental in China. Of these, 218 were recognized as separate species, while 489 were described as varieties and forms. These taxa were included across 48 genera. Genera with the most taxa described include *Pinnularia* (138), *Naviculasensu lato* (98), *Cymbellasensu lato* (56) and *Nitzschia* (43) and *Gomphonema* (39).

**Table 1.** Number of taxa described, Cumulative number of described taxa and number of publications in which new taxa were described from freshwaters in China, 1848–2019.

	Number of Taxa	Cumulative	Publications
1848–1867	9	9	2
1868–1887	0	9	0
1888–1907	28	37	1
1908–1927	57	94	2
1928–1947	355	449	16
1948–1967	8	457	2
1968–1987	189	646	13
1988–1999	61	707	17
2000–2019	421	1128	99

The data for this period were organized into 20-year intervals (except the last period) and show some interesting trends. The first works in which new taxa were described were penned by Europeans working alone. This included Ehrenberg being the first in 1848 and then again in 1854, but after that more than 50 years went by before another publication that included a new species described was produced. In 1906 Mereschkowsky studies diatoms from Tibet, and in 1922 Hustedt worked on diatoms from Tibet and the northwestern part of China. Starting in the late 1920’s, through the 1940’s, the description of continental diatom taxa was dominated by Boris V. Skvortzov. Williams et al. (2016) have provided insights into the life and work of Skvortzov, and Gololobova (2012) has created a checklist of the taxa described by him. Unlike his predecessors who described continental diatoms from China, Skvortzov actually lived and worked in China (though he studied from many parts of Asia, from the Philippines, Russia and to India, and further afield, and received samples from many individuals). His base was in Harbin, in Heilongjiang Province, in the northeastern part of the country. Thus, while species he described were from many areas and diverse ecosystems across China, many of the taxa described were from the northeastern part of the country. Skvortzov trained students in Harbin, and later work on diatom taxonomy in China has been carried out by three generations of scientists who can trace their academic lineages back to him. During the same time period as Skvortzov was describing many taxa, some Europeans also contributed to our knowledge of new continental diatoms, such as Skuja (1937) and Voigt (1942a, b).

In the period following Skvortzov’s large work published in 1946, only one other publication appeared (in which 8 taxa were described), until another large work on the continental diatoms of China was published by Skvortzov in 1976. From 1950 to 1976 was a period of dramatic challenges and cultural change in China. The first occurred with the severe famine that hit the country in 1950, lasting three years. The impacts of that famine led to the deaths of tens of millions of people, and this had lasting impacts on society for many years afterwards. In addition, the Cultural Revolution, in part a reaction to the great famine, also had negative impacts on life in China, and those impacts on academics are well-documented. Thus, with the few publications produced in this time period within the narrow discipline of diatom taxonomy and biodiversity discovery, we can see the impacts of natural disasters, economic decline and political and cultural change on the output and continuation of scientific research and training.

Skvortzov left China during the cultural revolution, and ended up in Sao Paulo, Brazil. While there, he published two large works in which nearly 140 taxa were described from Chinese continental (Skvortzov 1976a, b). These were to be his last works on the topic of continental diatoms from China while he was alive. His collections have never been found (See Williams et al. 2016).

Despite his death, and the uncertainty regarding his collections, Skvortzov’s legacy lives on with the works of his students, especially Professor Bao (who is currently in Harbin) and Professor Qi (who is currently in Guangzhou), both of whom are officially retired, as well as Professor Zhang, previously of Jinan University in Guangzhou (now deceased). All of these scientists ended up forming collaborations with scientists in the USA, with C.W. Reimer at the Academy of Natural Sciences of Philadelphia (who visited Harbin and hosted Bao, Qi and Zhang in Philadelphia) and E.F. Stoermer at the University of Michigan. Professor Qi visited Reimer at the Academy in Philadelphia while attending the International Diatom Symposium there in 1982 and stayed with Stoermer at his home in Ann Arbor, while on an extended trip to the USA in 1984. These connections yielded published collaborative works (Qi et al. 1984; Stoermer et al. 1986; Bao and Reimer 1992).

In the latter part of the 20^th^ century, we see important floristic works being published on the diatoms from Tibet, Yunnan and other localities, and some emphasis on freshwater fossil diatoms by Chinese researchers. In these books and papers, a new generation of scientists had come on the scene, and there was the initiation of an important series focused on documenting the freshwater diatom flora of the country (“Flora Algarum Sinicarum Aquae Dulcis” Qi 1995; Qi and Li 2004; Li and Qi 2010; Shi 2004, 2013; Wang and You 2018).

### The recent period (2000–2019)

In the 20-year time period, from 2000 to 2019, a total of 421 taxa, consisting of 252 species and 169 subspecific taxa across 67 genera have been described from continental waters in China (Appendix 2; Table 2). In addition, 6 new genera have been described. The breadth of lineages represented in these works during this period is remarkable, since several groups of “centric” and “araphid” diatoms have been described, as well as taxa among the major raphid lineages (Eunotiales, Bacillariales, Naviculales, Cymbellales, Rhopalodioid and Surirellales) are all included. For example, new genera of centric diatoms include *Edtheriotia* and the new genus of araphid diatoms is represented by *Tibetiella*. Raphid genera are included in the Eunotiales (*Sinoperonia*), Naviculales (*Sichuaniella*, *Pseudofallacia*) and Cymbellales (*Gomphosinica*). New species can be found among the centrics (in the genera *Cyclotella*, *Edtheriotia*, *Urosolenia*, *Orthoseira*, and *Melosira*), araphids (*Fragilaria*, *Tabularia*, *Diatoma* and *Tetracyclus*) and across the raphid diatoms, including the Eunotiales (*Eunotia*), Bacillariales (*Simonsenia*, *Achnanthes*), Monoraphids (*Achnanthidium*, *Platessa*), Naviculoids (*Germainiella*, *Neidium*, *Pinnularia. Muelleria*), Cymbelloids (*Cymbella*, *Delicata*), Rhopalodioids (*Epithemia*) and Surirelloids (*Cymatopleura*, *Surirella*). Please note that *Achnanthes*, though monoraphid, has been shown to be more closely related to members of the Bacillariales (Bruder and Medlin 2008); the monoraphid condition has evolved several times in the raphid diatom lineage (Kociolek et al. 2019a). Genera with the most species described in this period include *Pinnularia* (76), *Gomphonema*, (57), *Cymbella* (36), *Neidium* (22), *Amphora* (23).

**Table 2.** Number of taxa described, cumulative number of taxa, and number of publications in which taxa were described of freshwater diatoms in China, 2000–2019.

**Year**	**Number of taxa described**	**Cumulative number of described taxa**	**Number of Publications**
2000	2	2	1
2001	0	2	0
2002	1	3	1
2003	8	11	3
2004	10	21	2
2005	1	22	1
2006	1	23	1
2007	1	24	1
2008	1	25	1
2009	4	29	3
2010	8	37	6
2011	1	38	1
2012	209	247	3
2013	28	275	7
2014	17	292	7
2015	14	306	9
2016	11	317	9
2017	36	353	13
2018	38	391	15
2019	30	421	15
TOTAL	421		99

There is also great geographic breadth represented in these studies, with taxa being described in the northwestern portion of the country, Tibet and Yunnan, across the biodiverse regions of the karst belt extending from Yunnan to Guizhou, the central part of China, and from Hainan to the extreme Northeast.

Since 2000, there have been floristic studies that have yielded description of new taxa, such as Zhu and Chen’s (2000) tome on the diatoms of Tibet, as well as revisionary work, such as Shi’s (2004) study on gomphonemoid diatoms. While these were published in Chinese, the trend has been in more recent years for works to be published in English-language journals, such as Phytotaxa, Phycologia, Fottea, Cryptogamie: Algologie, Nova Hedwigia, and Diatom Research. Some research continues to be published in several Chinese-language journals as well.

In 2012, Kulikovskiy et al. included a paper offered by Gololobova and Kulikovskiy where they traced a manuscript submitted in the 1960’s by Skvortzov to Dr. Proschkina-Lavrenko in Moscow for publication. but the paper was, for unknown reasons, never published. In this paper, which has been published in the 23^rd^ volume of Iconographia Diatomologica, Skvortzov presents 445 taxa, that represent either new names, transfers or new taxon descriptions. This work includes taxa from India, China, Philippines, Japan, Korea, Australia and even Cuba. For the purposes of the current work, 208 of the taxa were newly described from China, and recorded for the year of publication (2012) even though the work was submitted 4 decades previously. These new taxon descriptions were not validly published (they lacked designation of type specimens) but are included here since they represent the identification and publication of new taxa in China.

In total, since 2000, the 421 described taxa were included in nearly 100 published books and papers (Table 2), nearly twice the number of publications than was published in the preceding 150 years. In some of the recent studies, observations have verified the continued presence of endemics described in earlier works (e.g. *Gomphonema eminens* Skuja in Yunnan; Liu et al. 2020; several species of *Pinnularia* from the Great A’er Mountains; Liu et al. 2018; and species of centric diatoms from Yunnan), though the population sizes of these endemic taxa are reported to be declining (Li et al. 2012b).

The degree of collaboration between Chinese researchers within and between institutions, and the inclusion of students in these works, are both striking and a demonstration that this area of research will have a fruitful period of work ahead. Amongst the senior and corresponding authors of these papers we see the impact of Skvortzov, whose academic grandchildren and great-grandchildren working on freshwater diatoms are now in Shanghai, Taiyuan, and Harbin. There are also well-established labs in Beijing, Shanghai, Kunming, Nanjing, and Jishou, whose focus may include the study of ecological and palaeoecological interpretation, as well as biodiversity discovery and description. Collaborations with non-Chinese scientists is also hallmark of this most recent era, with partners joining in these works from the U.K., Spain, Macedonia, Germany, Luxembourg, Belgium, Canada, and the USA.

## Discussion: A look ahead

While there has been a tremendous amount of work done to document the freshwater diatom flora of China, there are still many areas across the country that await initial or additional in-depth study. Some of these areas include, Yunnan Province, the vast karst region across Yunnan/Guangxi/Guizhou provinces, the subtropical southern part of the country, Xinjiang Autonomous Region, and Tibet, to name a few. In these regions are the two biodiversity hotspots the are fully in China (Myers et al. 2000; CEPF 2019). Also, the two longest rivers in China, The Yangtze River and Yellow River, have had isolated studies, but not comprehensive analyses. There exist specialized habitats such as also high mountain ranges, waterfalls, and hot springs, to name a few, where more intensive studies are warranted.

Perhaps one of the most challenging projects, with the potential of having the least “impact” (in the way most universities or state labs would assess that notion), but the greatest impact on the discipline, would be the typification of the diatom taxa described by Skvortzov. With the location of his collection uncertain (several generations of curators have attempted to track the collection to universities and institutes in China, Russia, Brazil, and Scandanavia), it seems likely that the collection has been lost. The challenge would not only be the large number of taxa he described. There would be a huge challenge to find specimens to designate as neotypes for his taxa, or to designate illustrations of his as epitypes. If neotypification was chosen, it would present many challenges, especially in situations where several varieties or forms were dissected from the same species, or for the large number of taxa described in his 1976 and 2012 papers, where the illustrations are of a quality that might not facilitate making positive identifications. It also appears that the collections of Chen and Zhu have been lost, and typification of their taxa will also be an important activity for taxonomists.

The loss of several important collections in China is not restricted to that country. Collections have been discarded by many universities and research institutes across the world. Currently, China does not have a national diatom collection. Such a repository might be useful in the future, as the significant, current activities of collection-building and biodiversity discovery and description, which appears to still be in a log growth phase (see Fig. 1). The fate of the collections that have been established and blossomed in a single generation in Shanghai, Harbin, Taiyuan, Beijing and other labs will always be tenuous. Having a national collection would potentially provide a repository for the country to serve future generations of scientists.

Although there is tremendous described diversity in the continental diatom flora of China, and it is likely that there is still much to do to achieve a more comprehensive knowledge of that flora, the number of strains of continental diatoms in GenBank traceable to a source in China is modest. In fact, this is all the more surprising since some groups of continental diatoms have a tremendous diversity in China, and some endemic genera in Asia have representatives in the Chinese flora (Kociolek 2019). Some of these groups include the Thalassiosiraceae and Cymbellales. In the latter group, the only endemic genera known for that lineage worldwide are from Asia and include species from China (e.g. Zhang et al. 2018). Workflows and resources will need to be developed so that the number of molecular sequences generated from Chinese taxa are commensurate with the diversity and unique nature of the flora.

## Appendix 1

Taxa of continental diatoms described from China, 1848–1999.

Achnanthes affinis var. biseriata Skvortzov 1938c

*Achnanthes cucurbita* Skvortzov 1935

*Achnanthes fragilis* Skvortzov 1938b

*Achnanthes fukiensis* Skvortzov 1929a

*Achnanthes girinensis* Skvortzov 1935

*Achnanthes guizhouensis* Chen and Zhu 1994

*Achnanthes hedini* Hustedt 1922

*Achnanthes hankensis* Skvortzov 1929c

*Achnanthes himalayensis* Jao and Zhu in Jao, Zhu and Lee 1974

Achnanthes inflata var. sinica Skvortzov 1929a

*Achnanthes kansouensis* Skvortzov 1935

Achnanthes linearis var. kankouensis Skvortzov 1935

Achnanthes linearis f. minuta Skvortzov 1935

*Achnanthes medioconvexa* Zhu and Chen 1996

*Achnanthes mesoconstricta* Zhu and Chen 1996

Achnanthes minutissima var. constricta Skvortzov 1935

*Achnanthes pamirensis* Hustedt 1922

*Achnanthes pinnata* Hustedt 1922

Achnanthes schmidtiana var. tibetica Jao and Zhu in Jao, Zhu and Lee 1974

*Achnanthes sublinearis* Skvortzov 1938c

Achnanthes sublinearis var. complexa Skvortzov 1938c

Achnanthes sublinearis var. elliptica Skvortzov 1938c

*Achnanthes tibetica* Jao 1964

Actinella brasiliensis var. curta Skvortzov 1929c

*Actinella miocenica* Li 1988

Amphiprora medulica var. sinensis Skvortzov 1927

*
Amphiraphia* Chen and Zhu 1983

*Amphiraphia xizangensis* Chen and Zhu 1983

Amphiraphia xizangensis var. major Chen and Zhu 1983

Amphora angusta var. sinensis Skvortzov 1927

*Amphora asiatica* Skvortzov 1935

Amphora dalaica var. hinganica Skvortzov 1976b

Amphora dalaica var. oculata Skvortzov 1976b

Amphora delicatissima f. sinica Skvortzov 1935

*Amphora geniculata* Hustedt 1922

*Amphora ostenfeldii* Hustedt 1922

Amphora ovalis f. mongolica Skvortzov 1930

*Amphora reniformis* Guo, Xie and Li 1997

Anomoeoneis polygramma var. rhomboides Jao 1964

Anomoeoneis polygramma var. tibetica Jao 1964

*Aulacoseira dianchiensis* Yang, Stoermer and Kociolek 1994

Caloneis bacillum f. latilanceolatum Zhu and Chen 1995

*Caloneis chansiensis* Skvortzov 1935

Caloneis fasciata var. pekinensis Skvortzov 1928a

Caloneis holstii var. tibetica Jao 1964

*Caloneis hunanensis* Chen and Zhu 1989

Caloneis lepidula var. angustata Skvortzov 1976a

Caloneis patagonica var. sinica Skvortzov 1938c

Caloneis schroderi var. densestriata Skvortzov 1976a

Caloneis schumanniana var. biconstricta f. minor Zhu and Chen 1995

Caloneis silicula var. hankensis Skvortzov 1929c

Caloneis schumanniana f. gracilis Skvortzov 1935

Caloneis silicula var. hinganica Skvortzov 1976a

*Caloneis sphagnicola* Skvortzov 1938b

Ceratoneis arcus var. orientalis Skuja 1937

Cocconeis placentula var. rotunda Skvortzov 1928a

*Coscinodiscus sinicus* Skvortzov 1946

*Cyclotella asterocostata* Lin, Xie & Cai in Xie et al. 1985

Cyclotella asterocostata var. borealis Xie and Cai 1985

Cyclotella asterocostata var. striata Chen 1987

*Cyclotella curvistriata* Chen and Zhu 1985

*Cyclotella florida* Voigt 1942

*Cyclotella hinganica* Skvortzov 1976a

*Cyclotella hubeiana* Chen and Zhu 1985

Cyclotella kuetzingiana var. hankensis Skvortzov 1929b

*Cyclotella lacunarum* Hustedt 1922

Cyclotella meneghiniana var. hankiensis Skvortzov 1929b

Cyclotella meneghiniana var. hinganica Skvortzov 1976a

*Cyclotella miyiensis* Qi and Yang 1985

*Cyclotella obliquata* Qi and Yang 1985

*Cyclotella rhomboideo-elliptica* Skuja 1937

Cyclotella rhomboideo-elliptica var. rounda Qi and Yang 1985

*Cyclotella shanxiensis* Xie and Qi 1984

*Cyclotella tibetana* Hustedt 1922

*Cymatopleura sinensis* Skvortzov 1927

Cymatopleura solea var. hankensis Skvortzov 1929c

Cymbella affinis var. elegans Mereschkowsky 1906

Cymbella amphioxys var. asiatica Skvortzow 1938e

*Cymbella amoyensis* Voigt 1942

Cymbella angustata var. hinganica Skvortzov 1976b

Cymbella aspera var. elongata Skvortzov 1928b

Cymbella aspera var. fossilis Skvortzov 1937

Cymbella aspera var. intermedia Skvortzov 1929c

Cymbella aspera var. manschurica Skvortzov 1928b

Cymbella aspera var. shantungensis Voigt 1942

Cymbella australica var. hankensis Skvortzov 1929c

Cymbella austriaca var. hankensis Skvortzov 1929c

*Cymbella cantonensis* Voigt 1942

Cymbella cantonensis var. obtusa Voigt 1942

Cymbella cesatii var. asiatica Skvortzov 1938b

Cymbella cistula var. asiatica Mereschkowsky 1906

Cymbella cistula var. heterostriata Mereschkowsky 1906

Cymbella cistula var. hinganensis Skvortzov 1928b

Cymbella cistula var. manschurica Skvortzov 1928b

Cymbella cistula var. recta Shi 1991

Cymbella cistula var. rotundata Voigt 1942

Cymbella cistula var. woosungensis Voigt 1942

Cymbella delicatula var. capitata Skvortzov 1935

Cymbella delicatula var. fasciata Voigt 1942

Cymbella delicatula var. magna Chen & Zhu in Zhu & Chen, 1994

Cymbella ehrenbergii var. apiculata Skvortzov 1976b

Cymbella ehrenbergii var. hankensis Skvortzov 1929c

*Cymbella globosa* Voigt 1942

Cymbella gracilis var. arcuata Voigt 1942

Cymbella gracilis var. arcuata Skvortzov 1976b

Cymbella gracilis f. crassiostriata Skvortzov 1976b

Cymbella gracilis var. kansouensis Skvortzov 1935

Cymbella gracilis f. sphagnicola Skvortzow 1938e

*Cymbella heteropleura* var. hinganicaSkvortzov 1976b

Cymbella lanceolata var. grossepunctata Skvortzov 1976b

*Cymbella jianghanensis* Shi 1991

*Cymbella jilinensis* Huang in Huang et al, 1983

*Cymbella jolmolungenesis* C.C. Jao & Y.Y. Lee in C.C. Zao, H.Z. Zhu & Y.Y. Lee 1974

Cymbella lacustris var. subtropica Voigt 1942

Cymbella lata var. sinica Skvortzov 1935

*Cymbella muralis* Skvortzov 1937

Cymbella naviculiformis f. constricta Skvortzov 1938d

Cymbella naviculiformis var. stauroptera Voigt 1942

*Cymbella pavlovi* Skvortzov 1938b

Cymbella perpusilla f. elongata Skvortzow 1938e

Cymbella ruttneri var. liaotungensis Skvortzov 1946

Cymbella signata var. chinensis Skvortzov 1929a

*Cymbella sinica* Skvortzov 1938c

Cymbella sinica var. miyiensis Qi and Yang 1985

Cymbella stuxbergii var. tumida Skvortzov 1938c

*Cymbella tenuistriata* Shi 1991

*Cymbella tibetana* Hustedt 1922

Cymbella tumidula f. recta Skvortzov 1938c

Cymbella turgida var. hinganica Skvortzov 1976b

Cymbella ventricosa var. major Skvortzov 1929c

Cymbella ventricosa var. pekinensis Skvortzov 1929a

Cymbella ventricosa f. major Mereschkowsky 1906

Denticula elegans var. hinganica Skvortzov 1976b

*Diatoma shenonngia* Zhang and Qi 1994

Diploneis elliptica var. hankae Skvortzov1929b

Diploneis elliptica var. mongolica Mereschkowsky 1906

Diploneis finnica var. sinica Skvortzov 1929a

*Diploneis lijingensis* Huang in Huang et al. 1998

*Diploneis rupestris* Skvortzov 1938a

*Discoplea atmosphaerica* Ehrenberg 1848

*Discoplea sinensis* Ehrenberg 1848

Epithemia hyndmanii var. chinensis Skvortzov 1929a

Epithemia zebra var. hankensis Skvortzov 1929c

*Eucocconeis hinganica* Skvortzov 1976a

*Eunotia anhuiensis* J.Yang 1995

Eunotia arcus var. bindulata Skvortzov 1976a

Eunotia arcus var. crassistriata Skvortzov 1976a

Eunotia arcus var. hinganica Skvortzov 1976a

Eunotia arcus var. triundulata Skvortzov 1976a

*Eunotia asiatica* Skvortzow 1938e

Eunotia asiatica var. interrupta Skvortzow 1938e

Eunotia bigibba var. rupestris Skvortzov 1938a

Eunotia clevei var. sinica Skvortzov 1929a

Eunotia faba var. lunata Skvortzov 1976a

Eunotia faba var. minor Skvortzov 1976a

Eunotia formica var. elongata Skvortzov 1929c

Eunotia fragilariodes var. elongata Skvortzov 1929b

Eunotia gracilis var. densestriata Skvortzov 1976a

*Eunotia hainanensis* Zhang et Qi 1993

*Eunotia hinganica* Skvortzov 1976a

Eunotia lunaris var. undulata Skvortzov 1976a

Eunotia major var. asiatica Skvortzov 1929a

Eunotia major var. hankensis Skvortzov 1929b

Eunotia monodon var. asiatica Skvortzov 1936

Eunotia asiatica var. interrupta Skvortzow 1938e

Eunotia parallela f. asiatica Skvortzov 1938b

Eunotia parallela var. hinganica Skvortzov 1976a

Eunotia pectinalis var. chinensis Skvortzov 1929b

*Eunotia plicata* Jao 1964

Eunotia praerupta var. tibetica Mereschkowsky 1906

Eunotia sudetica var. hankensis Skvortzov 1929b

Eunotia suecica f. hankensis Skvortzov 1929c

Eunotia suecica var. hinganica Skvortzov 1976a

Eunotia suecica var. simplex Skvortzov 1976a

Eunotia tautoniensis var. hankensis Skvortzov 1929b

Eunotia tautoniensis var. undulata Skvortzov 1929c

*Fragilaria asiatica* Hustedt 1922

Fragilaria brevistriata var. bigibba Jao 1964

Fragilaria brevistriata var. tibetica Mereschkowsky 1906

*Fragilaria curvata* Skvortzov 1935

*Fragilaria hainanensis* Zhang et Qi 1994

*Fragilaria hinganensis* Skvortzov 1928b

Fragilaria hinganensis var. longissima Skvortzov 1928b

*Fragilaria indigema* Skvortzov 1976a

Fragilaria lapponica var. lanceolata Zhang and Qi 1994

Fragilaria leptostauron var. hainanensis Zhang in Zhang and Qi 1994

*Fragilaria mesotyla* Ehrenberg 1848

*Fragilaria shangdongensis* Li 1982

*Fragilaria subtriundulata* Li 1999

Fragilaria virescens var. acicularis Skvortzov 1976a

Fragilaria virescens var. obtusa Skvortzov 1976a

Fragilaria virescens var. restratus Skvortzov 1976a

*Frustulia chinensis* Skvortzov 1929b

Frustulia vulgaris var. asiatica Skvortzov 1928b

Frustulia vulgaris var. constricta Skvortzov 1929b

Frustulia vulgaris var. muscosa Skvortzov 1937

Frustulia vulgaris var. rupestris Skvortzov 1938a

*Gloeonema sinense* Ehrenberg 1848

Gomphonema acuminatum f. elongatum Skvortzov 1929c

Gomphonema acuminatum var. sinica Skvortzov 1935

Gomphonema acuminatum var. tibeticum Jao 1964

Gomphonema augur f. hankensis Skvortzov 1929c

Gomphonema augur f. orientalis Skvortzov 1929c

Gomphonema augur var. sinica Skvortzov 1946

*Gomphonema changyangicum* Li et al 1999

Gomphonema clevei f. heterovalvata Voigt 1942

Gomphonema clevei var. sinensis Voigt 1942

Gomphonema constrictum var. amphicephala Mereschkowsky 1906

Gomphonema constrictum var. elongata Skvortzov 1929c

Gomphonema constrictum var. hankensis Skvortzov 1929c

*Gomphonema eminens* Skuja 1937

*Gomphonema hinganicum* Skvortzov 1976b

Gomphonema hinganicum var. apiculatum Skvortzov 1976b

*Gomphonema hedini* Hustedt 1922

Gomphonema heideni var. sinica Skvortzov 1938c

*Gomphonema hubeicum* Li et al. 1999

Gomphonema instabilis f. wangii Bao and Reimer 1992

*Gomphonema interruptum* Chen and Zhu 1994

*Gomphonema kasnakowi* Mereschkowsky 1906

Gomphonema kasnakowi var. distincta Skvortzov 1938c

*Gomphonema laojunshanensis* Li, Kociolek and Metzeltin 2010b

*Gomphonema licenti* Skvortzov 1935

Gomphonema licenti var. curta Skvortzov 1935

Gomphonema longiceps var. australica Skvortzov 1976b

Gomphonema longiceps f. minuta Skvortzow 1938e

Gomphonema montanum f. hankensis Skvortzov 1929c

Gomphonema olivaceum var. tibetica Mereschkowsky 1906

Gomphonema parvulum var. deserta Skvortzov 1935

Gomphonema parvulum var. sinica Skvortzov 1935

Gomphonema puiggarianum var. sinica Skvortzov 1929b

Gomphonema ruttneri var. liaotungensis Skvortzov 1946

*Gomphonema sinica* Skvortzov 1929b

Gomphonema sphaerophorum var. asiatica Skvortzov 1929a

Gomphonema sphaerophorum var. densestriatum Zhu & Chen, 1994

Gomphonema subclavatum var. hankensis Skvortzov 1929c

Gomphonema tergestinum var. shantungensis Skvorttzow 1937

Gomphonema tropicale var. nonpunctatum Shi 1991

*Gomphonema xiphoidea* Skvortzov 1976b

Gyrosigma attenuatum var. asiatica Skvortzov 1938c

*Gyrosigma hankensis* Skvortzov 1929c

Gyrosigma spencerii var. sinica Skvortzov 1929b

Hantzschia amphioxys var. compacta Hustedt 1922

Hantzschia amphioxys f. hankensis Skvortzov 1929c

Hantzschia amphioxys var. hinganensis Skvortzov 1928b

Hantzschia amphioxys var. sinica Skvortzov 1946

Hantzschia elongata var. curta Skvortzov 1976b

Hantzschia elongata var. densestriata Skvortzov 1976b

Hantzschia elongata var. obtusa Skvortzov 1976b

Hantzschia elongata var. tenua Skvortzov 1976b

Mastogloia braunii var. sinensis Skvortzov 1927

Mastogloia grevillei var. sinica Skvortzov 1927

Mastogloia brunii var. sinensis Skvortzov 1927

Melosira brunii var. sinensis Skvortzov 1927

*Melosira cuculleta* Chen 1980

*Melosira hunanica* Chen & Zhu in Huang et al. 1998

Melosira irregularis var. hankensis Skvortzov 1929b

Melosira italica var. hankensis Skvortzov 1929b

*Melosira radiato-sinuata* Chen 1980

Melosira radiato-sinuata var. yunnanica Chen 1980

Melsoria roseana var. asiatica Skvortzov 1938a

Melosira roeseana var. epidendron f. spinosa Skvortzov 1938a

Melosira roseana f. spinosa Skvortzov 1938a

Melosira roeseana var. xizangensis Chen in Chen and Zhu 1995

*Melosira sinensis* Chen 1980

*Melosira soochowensis* Skvortzov 1946

Melosira young i var. tenuissima Skvortzov 1937

Meridion circulare var. subcapitatum Skvortzov 1976a

*Navicula aktinoides* Skuja 1937

Navicula americana f. hankensis Skvortzov 1929c

Navicula amphibola var. manschurica Skvortzov 1928b

*Navicula anhuiensis* Yang 1994

*Navicula argunensis* Skvortzov 1938d

Navicula bacillum var. elongata Skvortzov 1929c

Navicula bacillum var. hankensis Skvortzov 1929c

Navicula bacillum var. parallela Skvortzov 1938d

Navicula barentsii var. capitata Bao and Reimer 1992

*Navicula bigibba* Chen and Zhu 1994

Navicula brockmannii var. undulata Zhu and Chen 1996

Navicula bryophila var. paucistriata Zhu and Chen 1996

*Navicula cantonensis* Ehrenberg 1848

Navicula cari var. rostrata Skvortzov 1935

*Navicula chansiensis* Skvortzov 1935

*Navicula chinensis* Skvortzov 1929b

Navicula cincta var. kansouensis Skvortzov 1935

Navicula cincta var. minuta Skvortzov 1937

Navicula cincta var. sphagnicola Skvortzow 1938e

Navicula cryptocephala var. australis Skuja 1937

Navicula cryptocephala var. hankensis Skvortzov 1929c

Navicula cuspidata f. craticularis Skvortzov 1929c

Navicula cuspidata var. hankae Skvortzov 1929c

Navicula cuspidata var. tibetica Jao 1964

Navicula exigua var. signata Skvortzov 1935

Navicula exigua var. sinica Skvortzov 1935

*Navicula fukiensis* Skvortzov 1929b

Navicula gastrum var. hankensis Skvortzov 1929c

Navicula gastrum var. limnetica Skvortzov 1929c

Navicula gastrum var. mongolica Skvortzov 1928c

*Navicula hangchowensis* Skvortzov 1937

*Navicula hankae* Skvortzov 1929c

*Navicula hedini* Hustedt 1922

*Navicula hunanensis* Chen and Zhu 1994

Navicula incerta f. asiatica Skvortzov 1935

*Navicula jkarii* Skvortzov 1929c

*Naviculaa kalganica* Skvortzov 1935

Navicula kotschyi f. hinganica Skvortzov 1976a

Navicula kotschyi var. rupestris Skvortzov 1938a

*Navicula kovalchookiana* Skvortzov 1937

Navicula laevissima var. lanceolata Skvortzov 1938a

Navicula laevissima var. ovata Skvortzov 1937

Navicula laevissima var. robusta Skvortzov 1938a

Navicula lagerheimii var. capitata Skvortzov 1937

Navicula lagerheimii var. lanceolata Skvortzov 1938a

Navicula lagerheimii var. ovata Skvortzov 1937

Navicula lagerheimii var. robusta Skvortzov 1938a

Navicula lambda var. recta Skvortzov 1929b

Navicula lambda var. sinica Skvortzov 1935

*Navicula licenti* Skvortzov 1935

Navicula menisculus var. sinica Skvortzov 1935

*Navicula mongolica* Skvortzov 1935

*Navicula multipunctata* Chen and Zhu 1994

*Navicula muscosa* Skvortzow 1938e

Navicula mutica var. rhombica Skvortzov 1938a

*Navicula mutica* var. sinicaYang 1994

Navicula nivalis var. chinensis Skvortzov 1929b

Navicula oblonga var. linearis Mereschkowsky 1906

*Navicula ocellata* Skvortzov 1938a

Navicula ocellata var. polymorpha Skvortzov 1938a

*Navicula parva* Skvortzov 1935

Navicula peregrina var. asiatica Skvortzov 1929c

Navicula peregrina f. curta Skvortzov 1929c

Navicula peregrina var. hankensis Skvortzov 1929c

Navicula peregrina var. lanceolata Skvortzov 1929c

Navicula peregrina var. minuta Skvortzov 1929c

Navicula peregrina var. sinica Skvortzov 1938c

*Navicula perpusilloides* Chen and Zhu 1994

*Navicula praegnans* Skuja 1937

*Navicula pseudolinearis* Hustedt 1922

*Navicula pseudoseminulum* Skvortzov 1935

*Navicula puncticulata* Skvortzov 1976a

Navicula pupula var. elongata Skvortzov 1976a

Navicula pupula var. sinica Skvortzov 1935

Navicula radiosa var. hankensis Skvortzov 1929c

Navicula radiosa var. manschurica Skvortzov 1928b

Navicula rhynchocephala var. hankensis Skvortzov 1929c

Navicula rhynchocephala f. hankensis Skvortzov 1929c

Navicula rhynchocephala var. tenua Skvortzov 1938c

Navicula semen var. lineata Skvortzov 1976a

*Navicula seminuda* C. Jao & Y.Y.Lee in Jao, Zhu and Lee 1974

Navicula seposita var. major Zhu and Chen 1994

*Navicula setchwanensis* Skuja 1937

*Navicula siberica* Skvortzov 1929c

*Navicula sinensis* Ehrenberg 1848

Navicula soehrensis var. parallela Skvortzow 1938e

*Navicula soochowensis* Skvortzov 1946

Navicula soodensis var. mongolica Skvortzov 1935

*Navicula stagna* Chen and Zhu 1994

*Navicula subrhombica* Hustedt 1922

Navicula subvitrea var. maxima Skvortzov 1938c

*Navicula tientsinensis* Skvortzov 1927

*Navicula tytthocephala* Skvortzov 1976a

Navicula viridula var. alisoviana Skvortzov 1929c

Navicula viridula var. hankensis Skvortzov 1929c

Navicula viridula var. pamirensis Hustedt 1922

Navicula viridula var. rostrata Skvortzov 1938d

Navicula viridula var. pamirensis Hustedt 1922

Neidium affine f. manschurica Skvortzov 1928b

Neidium affine var. amphirhynchus f. manchuria Skvortzov 1928b

Neidium affine f. hankensis Skvortzov 1929

*Neidium bigibborum*Zhu and Chen 1995c

Neidium bisulcatum f. hankensis Skvortzov 1929c

Neidium bisulcatum f. latior Skvortzov 1976a

Neidium bisulcatum f. longissima Skvortzov 1976a

Neidium bisulcatum var. notata Mereschkowsky 1906

Neidium bisulcatum f. subcapitatum Skvortzov 1976a

*Neidium constrictum* Zhu and Chen 1995

*Neidium didelta* Hustedt 1922

*Neidium dilatatum* var. angustatumSkvortzov 1976a

*Neidium ellipticis* Zhu and Chen 1995

*Neidium ellipticum* Voigt 1942

*Neidium hankensis* Skvortzov 1929

Neidium hitchcockii f. hankensis Skvortzov 1929c

*Neidium hitchcockii* var. obliquestriatum Skvortzov 1935

Neidium iridis var. dissimilia Jao & Zhu in Jao, Zhu and Lee 1974

Neidium iridis f. hanganica Skvortzov 1976a

Neidium iridis var. orochenicum Skvortzov 1976a

Neidium kozlowi var. amphicephala Mereschkowsky 1906

Neidium kozlowi var. elliptica Mereschkowsky 1906

*Neidium kozlowi* var. elpatievskyi f. majus Zhu and Chen 1995

Neidium kozlowi var. hankensis Skvortzov 1929c

Neidium kozlowi var. parva Mereschkowsky 1906

*Neidium kozlowii* Mereschkowsky 1906

*Neidium manshuricum* Skvortzov 1946

Neidium maximum var. hankensis Skvortzov 1929c

*Neidium mirabile* Hustedt 1922

*Neidium oblongum* Zhu and Chen 1995

*Neidium punctulatum* Hustedt 1922

*Neidium radiosum* Voigt 1942

*Neidium rectum* Hustedt 1922

Nitzschia acuta var. hinganica Skvortzov 1976b

*Nitzschia amoyensis* Skvortzov 1929a

Nitzschia amphibia var. curta Skvortzov 1928a

*Nitzschia bacillariaeformis* Hustedt 1922

*Nitzschia bacilliformis* Hustedt 1922

Nitzschia bacilliformis var. elongata Skvortzov 1935

*Nitzschia bacillum* Hustedt 1922

Nitzschia bremensis var. sinica Skvortzov 1935

Nitzschiaa capitellata var. mongolica Skvortzov 1935

Nitzschia capitellata var. montana Skvortzov 1938b

*Nitzschia chinensis* Hustedt 1922

Nitzschia denticula var. elongata Mereschkowsky 1906

*Nitzschia denticulata* Skvortzov 1935

Nitzschia denticulata var. undulata Skvortzov 1935

*Nitzschia filia* Skvortzov 1976b

Nitzschia filia var. apiculata Skvortzov 1976b

Nitzschia frustulum var. asiatica Hustedt 1922

Nitzschia frustulum var. mongolica Skvortzov 1935

*Nitzschia gradifera* Hustedt 1922

*Nitzschia grigoriewi* Mereschkowsky 1906

*Nitzschia hankensis* Skvortzov 1929c

Nitzschia heufleriana var. asiatica Skvortzov 1929c

Nitzschia ignorata var. asiatica Skvortzov 1938b

Nitzschia heidenii var. pamirensis Boye Peterson 1930

*Nitzschia iugiformis* Hustedt 1922

*Nitzschia jugiformis* Hustedt 1922

*Nitzschia mongolica* Skvortzov 1935

*Nitzschia ostenfeldi* Hustedt 1922

Nitzschia palea var. gracilis Skvortzov 1935

Nitzschia palea var. hinganica Skvortzov 1976b

*Nitzschia pamirensis* Hustedt 1922

*Nitzschia pekinensis* Skvortzov 1928a

*Nitzschia pseudolinearis* Hustedt 1922

*Nitzschia regula* Hustedt 1922

Nitzschia regula f. pekinensis Skvortzov 1928a

Nitzschia rigida var. sinensis Skvortzov 1927

Nitzschia sigma var. serpentina Skvortzov 1927

Nitzschia sinuata var. constricta Chen and Zhu 1989

*Nitzschia subvitrea* Hustedt 1922

*Nitzschia tibetana* Hustedt 1922

*Nitzschia tingrica* Jao & Lee in Jao, Zhu and Lee 1974

*Nitzschia yunchengensis* Xie and Li 1994

Nitzschia zabelini var. hinganica Skvortzov 1976b

*Pliocaenicus cathayanus* G.Wang 1999

*Pliocaenicus jilinensis* G.Wang 1999

Pinnularia acrosphaeria f. hankensis Skvortzov 1929c

Pinnularia aestuari var. hinganica Skvortzov 1976b

Pinnularia alpina f. symmetrica Mereschkowsky 1906

*Pinnularia archaica* Skvortzov 1976a

Pinnularia bogotensis var. asiatica Skvortzov 1938b

Pinnularia bogotensis var. hankensis Skvortzov 1929a

Pinnularia brebissonii f. curta Skvortzov 1929a

Pinnularia cardinalis var. constricta Skvortzov 1976b

Pinnularia cardinalis var. fenestrata Skvortzov 1976b

Pinnularia cardinalis f. angustior Skvortzov 1976b

Pinnularia cardinalis f. hankensis Skvortzov 1929c

Pinnularia cardinalis var. hinganica Skvortzov 1976b

Pinnularia cardinalis f. minuta Skvortzov 1976b

*Pinnularia centropuncta* Skvortzov 1976a

Pinnularia centropuncta var. lineata Skvortzov 1976a

*Pinnularia cheng* Skvortzov 1946

*Pinnularia chinensis* Skvortzov 1929a

*Pinnularia cholnokyi* Skvortzov 1976b

Pinnularia cholnokyi var. unilateralis Skvortzov 1976b

*Pinnularia congeri* Skvortzov 1976b

Pinnularia distinguenda var. asiatica Skvortzov 1938b

Pinnularia distinguenda f. striolata Skvortzov 1938b

Pinnularia distinguenda var. sphagnalis Skvortzow 1938e

Pinnularia divergens var. continua Mereschkowsky 1906

Pinnularia divergens var. tumida Skvortzov 1976a

Pinnularia divergentissima var. capitata Hustedt 1922

Pinnularia divergentissima var. lata Skvortzow 1938e

Pinnularia dorgogostaiskii var. latior Skvortzov 1976b

Pinnularia episcopalis var. hankensis Skvortzov 1929c

Pinnularia episcopallis var. lineata Skvortzov 1976a

Pinnularia episcopalis var. manschurica Skvortzov 1928b

Pinnularia esox var. hinganica Skvortzov 1976b

*Pinnularia essentialis* Skvortzov 1976a

*Pinnularia fonticola* Hustedt 1922

*Pinnularia fritschiana* Skvortzov 1976b

Pinnularia gibba f. constricta Skvortzov 1938b

Pinnularia gibba f. polymorpha Skvortzow 1938e

Pinnularia gracillima var. hinganica Skvortzov 1976a

Pinnularia hartleyana f. minor Skvortzov 1946

*Pinnularia hedini* Hustedt 1922

*Pinnularia hinganica* Skvortzov 1976b

Pinnularia interrupta f. hankensis Skvortzov 1929c

Pinnularia interrrupta var. sinica Skvortzov 1935

Pinnularia isostauron var. orientalis Skvortzow 1938e

Pinnularia karelica var. subcapitata Skvortzow 1938e

*Pinnularia kisselewii* Skvortzov 1976b

Pinnularia kisselewii var. hinganica Skvortozw 1976b

Pinnularia kisselewii var. intermedia Skvortzov 1976b

Pinnularia kisselewii var. parallela Skvortzov 1976b

Pinnularia kisselewii var. subacuta Skvortzov 1976b

*Pinnularia lacushankae* Skvortzov 1946

Pinnularia lata var. hinganica Skvortzov 1976a

Pinnularia lata var. intermedia Skvortzov 1976a

*Pinnularia latofasciata* Skvortzov 1946

Pinnularia legumen var. hinganica Skvortzov 1976a

Pinnularia leptosoma var. hinganica Skvortzov 1976a

Pinnularia liouana var. hinganica Skvortzov 1976a

*Pinnularia major* Skvortzov 1928b

Pinnularia major f. hankensis Skvortzov 1929c

Pinnularia major f. hinganica Skvortzov 1976a

Pinnularia major var. shantungensis Skvortzov 1937

Pinnularia mesolepta var. angusta f. sinica Skvortzov 1929a

Pinnularia mesolepta f. hankensis Skvortzov 1929c

Pinnularia mesolepta f. hinganica Skvortzov 1976a

Pinnularia mesolepta f. sinica Skvortzov 1929a

Pinnularia mesolepta f. subundulata Skvortzov 1929c

Pinnularia meyerii var. hinganica Skvortzov 1976b

Pinnularia microstauron f. curta Skvortzov 1929c

Pinnularia molaris var. asiatica Skvortzov 1938b

Pinnularia molaris var. constricta Skvortzov 1976a

Pinnularia molaris var. hinganica Skvortzov 1976a

*Pinnularia mongolica* Skvortzov 1935

Pinnularia mongolica var. lanceolata Skvortzov 1946

Pinnularia montana var. sinica Skvortzov 1929a

Pinnularia nobilis var. constricta Skvortzov 1976b

Pinnularia nobilis var. densestriata Skvortzov 1976b

Pinnularia nobilis var. distincta Skvortzow 1938e

Pinnularia nobilis var. gracillima Skvortzov 1976b

Pinnularia nobilis var. manschurica Skvortzov 1928

Pinnularia nobilis f. minor Skvortzov 1976b

Pinnularia nobilis var. obesa Skvortzov 1976b

Pinnularia nobilis var. soochowensis Skvortzov 1946

Pinnularia nobilis var. triundulata Skvortozw 1946

Pinnularia nodosa var. hankensis Skvortzov 1929c

Pinnularia nodosa var. maakii Skvortzov 1929c

*Pinnularia patrickii* Skvortzov 1976b

Pinnularia patrickii var. angustata Skvortzov 1976b

Pinnularia patrickii var. interrupta Skvortzov 1976b

Pinnularia patrickii var. pandurinifomis Skvortzov 1976b

Pinnularia polyonca var. hinganica Skvortzov 1976a

Pinnularia polyonca var. major Skvortzov 1929c

Pinnularia polyonca var. sublinearis Skvortzov 1976a

*Pinnularia prima* Skvortzov 1976a

Pinnularia prima var. subcapitata Skvortzov 1976b

*Pinnularia reimerii* Skvortzov 1976b

Pinnularia reimerii var. interrupta Skvortzov 1976b

Pinnularia savanensis var. hinganica Skvortzov 1976a

Pinnularia savanensis var. ignota Skvortzov 1976a

*Pinnularia secunda* Skvortzov 1976b

Pinnularia selengensis var. hinganica Skvortzov 1976a

*Pinnularia sinomongolica* Skvortzov 1976a

Pinnularia sinomongolica var. angustior Skvortzov 1976a

*Pinnularia sphagnicola* Skvortzow 1938e

Pinnularia spitzbergensis var. hinganica Skvortzov 1976a

Pinnularia stauroptera var. chinensis Skvortzov 1929a

Pinnularia stauroptera f. hankensis Skvortzov 1929c

Pinnularia stauroptera var. recta Skvortzov 1929c

Pinnularia stauroptera f. subcapitata Skvortzov 1929c

Pinnularia stomatophora var. hinganica Skvortzov 1976a

Pinnularia streptoraphe var. asiatica Skvortzov 1938b

Pinnularia streptoraphe var. muscicola Skvortzov 1976b

*Pinnularia subborealis* Hustedt 1922

Pinnularia subcapitata f. constricta Skvortzov 1938b

Pinnularia subcapitata var. sinica Skvortzov 1935

Pinnularia subcapitata f. tenua Skvortzov 1938e

Pinnularia subsolaris var. asiatica Skvortzow 1938e

Pinnularia subsolaris var. interrupta Skvortzov 1935

*Pinnularia tibetana* Hustedt 1922

Pinnularia tibetana var. argunensis Skvortzov 1938d

Pinnularia tibetana var. stauroneiformis Skvortzov 1946

Pinnularia tibetana var. truncata Skvortzov 1946

*Pinnularia tibetica* Mereschkowsky 1906

*Pinnularia turizaninow* Skvortzov 1976b

Pinnularia viridis f. argunensis Skvortzov 1938d

Pinnularia viridis var. fasoiata Skvortzov 1976b

Pinnularia viridis f. hankensis Skvortzov 1929c

Pinnularia viridis var. hinganica Skvortzov 1976b

Pinnularia viridis f. hinganica Skvortzov 1976b

Pinnularia viridis var. jenisseyensis Skvortzov 1976b

Pinnularia viridis f. muscicola Skvortzov 1976b

Pinnularia viridis var. orientalis Skvortzow 1938e

Pinnularia viridis var. sinica Skvortzov 1946

Pinnularia viridis var. ussuriensis Skvortzov 1929c

*Pinnularia zabelini* Skvortzov 1976b

Pinnularia zabelini var. amurensis Skvortzov 1976b

Pinnularia zabelini var. dimidia Skvortzov 1976b

Pinnularia zabelini var. zeaana Skvortzov 1976b

Pinnularia zabelini var. interrupta Skvortzov 1976b

Pleurosigma spenceri var. sinensis Skvortzov 1927

Pleurosigma spenceri var. tientsinensis Skvortzov 1927

*
Porosularia* Skvortzov 1976b

*Porosularia amoyensis* Skvortzov 1976b

*Porosularia borgei* Skvortzov 1976b

*Porosularia calawayi* Skvortzov 1976b

Porosularia calawayi var. undulata Skvortzov 1976b

*Porosularia chowyiliangi* Skvortzov 1976b

*Porosularia handel-mazzettii* Skvortzov 1976b

*Porosularia jurilyi* Skvortzov 1976b

Porosularia jurilyi var. striata Skvortzov 1976b

*Porosularia kolbei* Skvortzov 1976b

*Porosularia lackeyi* Skvortzov 1976b

*Porosularia liouningyanii* Skvortzov 1976b

*Porosularia meisteri* Skvortzov 1976b

*Porosularia merrilli* Skvortzov 1976b

*Porosularia poretskyi* Skvortzov 1976b

*Poroularia poroidea* Skvortzov 1976b

*Porosularia pseudoviridis* Skvortzov 1976b

*Porosularia pulchra* Skvortzov 1976b

*Porosularia scheschukewii* Skvortzov 1976b

*Porosularia skujae* Skvortzov 1976b

Porosularia skujae var. unilateralis Skvortzov 1976b

*Porosularia striata* Skvortzov 1976b

*Porosularia subsalsa* Skvortzov 1976b

*Porosularia wislouchi* Skvortzov 1976b

Rhopalodia gibba var. gracilis Skvortzov 1976b

Rhopalodia gibba var. major Skvortzov 1928b

Rhopalodia gibberula f. mongolica Mereschkowsky 1906

Rhopalodia gibberula f. tibetica Mereschkowsky 1906

*Rhopalodia pseudogibba* Skvortzov 1976b

*Rhopalodia tibetica* Mereschkowsky 1906

*Schizostauron sorokninii* Mereschkowsky 1906

*Scoliopleura pavlovi* Skvortzov 1930

Stauroneis anceps var. hankensis Skvortzov 1929c

Stauroneis anceps var. oblonga Skvortzov 1929c

Stauroneis anceps var. orientalis Skvortzov 1929c

Stauroneis anceps var. hinganica Skvortzov 1976a

Stauroneis anceps var. kansouensis Skvortzov 1935

Stauroneis anceps var. ussuriensis Skvortzov 1929c

*Stauroneis chinensis* Skvortzov 1929b

Stauroenis gregori var. hankensis Skvortzov 1929c

*Stauroneis jimeiensis* Lin 1989

*Stauroneis laticeps* Hustedt 1922

*Stauroneis okamurae* Skvortzov 1929c

Stauroneis parvula var. rupestris Skvortzov 1937

Stauroneis phoenicenteron f. curta Skvortzov 1929c

Stauroneis phoenicenteron var. genuina Skvortzov 1928b

Stauroneis phoenicenteron var. hankensis Skvortzov 1929c

Stauroneis phoenicenteron f. hankensis Skvortzov 1929c

Stauroneis phoenicenteron var. oblongella Skvortzov 1929c

*Stauroneis rupestris* Skvortzov 1938a

*Stauroneis tibetica* Mereschkowsky 1906

*Stauroptera granulata* Ehrenberg 1848

*Stenopterobia sigmoidea* Skvortzov 1976

*Stephanodiscus sinensis* Ehrenberg 1854

*Stephanodiscus soochowensis* Skvortzov 1946

*Surirella alisoviana* Skvortzov 1929c

Surirella angusta var. amoyensis Skvortzov 1929b

Surirella angusta var. constricta Skvortzov 1929c

Surirella angusta var. curta Skvortzov 1929c

Surirella angusta var. elongata Skvortzov 1929c

Surirella angusta var. hankensis Skvortzov 1929b

Surirella angusta f. ovata Skvortzov 1929c

Surirella biseriata var. orientalis Skvortzov 1929c

Surirella biseriata var. ussuriensis Skvortzov 1929c

*Surirella borscowi* Mereschkowsky 1906

Surirella capronii var. hankensis Skvortzov 1929c

*Surirella chachinae* Skvortzov 1929c

Surirella didyma var. hinganica Skvortzov 1976b

Surirella elegans var. hankensis Skvortzov 1929c

*Surirella elliptica* Ehrenberg 1848

Surirella engleri var. hankensis Skvorttzow 1929c

*Surirella fukiensis* Skvortzov 1929a

*Surirella hinganica* Skvortzov 1976b

Surirella linearis var. vermifera Skvortzov 1938c

Surirella ovalis var. hankensis Skvortzov 1929c

Surirella ovalis f. tientsinensis Skvortzov 1927

Surirella ovata f. curta Skvortzov 1930

Surirella ovata f. mongolica Skvortzov 1930

Surirella patella var. hankensis Skvortzov 1929c

Surirella robusta var. hankensis Skvortzov 1929c

Surirella robusta var. manschurica Skvortzov 1928b

Surirella saxonica var. sinica Skvortzov 1929a

Surirella splendida var. hankensis Skvortzov 1929c

Surirella tenera var. hinganica Skvortzov 1976b

*Surirella tibetica* Mereschkowsky 1906

*Surirella tientsinensis* Skvortzov 1927

*Surirella ussuriensis* Skvortzov 1929c

Surirella ussuriensis var. elegans Skvortzov 1929c

Surirella ussuriensis var. elongata Skvortzov 1929c

Synedra affinis var. sinica Skvortzov 1935

Synedra amphicephala var. asiatica Skvortzov 1935

*Synedra licenti* Skvortzov 1935

Synedra mazamaensis var. changbaiensis Bao and Reimer 1992

Synedra rumpens var. sinica Skvortzov 1935

Synedra tenera var. sinica Skvortzov 1935

Synedra ulna f. constricta Skvortzov 1938c

Synedra ulna f. curta Skvortzov 1928c

Synedra ulna var. anhuiensis Yang 1990

Synedra ulna var. intermedia Mereschkowsky 1906

Synedra ulna var. lanceolata f. constricta Skvortzov 1938c

Synedra ulna var. mongolica Skvortzov 1928c

Synedra ulna var. tenuirostris Skvortzov 1938c

Synedra vaucheriae var. capitata Skvortzov 1938c

Tetracyclus celatom var. minor Li 1999

*Tetracyclus dunhuanensis* Li 1984

Tetracyclus ellipticus var. austrochinensis Zhang 1994

Tetracyclus ellipticus var. ovaliformis Li 1984

Tetracyclus ellipticus var. rostrata Li 1984

*Tetracyclus jaoi* Li 1984

*Tetracyclus minutus* Li 1999

*Tetracyclus mucronatus* Li 1999

*Tetracyclus navicularis* Li 1984

*Tetracyclus ovaliformis* Li 1984

*Tetracyclus radiatus* Li 1999

Tetracyclus rupestris var. subclypeatus Li 1999

*Tetracyclus shangduensis* Li 1984

*Tetracyclus sinensis* Li 1984

*Tetracyclus subclypeus* Li and Williams 1990

*Tetracyclus subdivisium* Williams and Li 1990

Tetracyclus subdivisium var. ellipticus Li 1999

Thalassiosira lacustris var. crassiospinua Cai et Xie in Xie and Cai 1981

Tropidoneis maxima var. sinensis Skvortzov 1927

*Tryblionella debilis* var. sinensisSkvortzov 1927

Tryblionella hantzschiana f. sinensis Skvortzov 1927

*Tryblionella tryblionella* Skvortzov 1929c

Tryblionella tryblionella f. hankensis Skvortzov 1929c

## Appendix 2

Listing of New Taxa Described from continental waters of China, 2000–2019. Genera are in bold.

*Achnanthes chinii* Skvortzov ex Gololobov & Kulikovskiy in Kulikovskiy et al. 2012

Achnanthes coarctata subsp. fukinensis Skvortzov ex Gololobov & Kulikovskiy in Kulikovskiy et al. 2012

*Achnanthes dalaica* Skvortzov ex Gololobov & Kulikovskiy in Kulikovskiy et al. 2012

*Achnanthes drepanocladoides* Skvortzov ex Gololobov & Kulikovskiy in Kulikovskiy et al. 2012

Achnanthes drepanocladoides var. fukinensis Skvortzov ex Gololobov & Kulikovskiy in Kulikovskiy et al. 2012

Achnanthes gracillima var. sinica Skvortzov ex Gololobov & Kulikovskiy in Kulikovskiy et al. 2012

Achnanthes hankiana var. sinica Skvortzov ex Gololobov & Kulikovskiy in Kulikovskiy et al. 2012

Achnanthes kansouensis var. septentrionalis Skvortzov ex Gololobov & Kulikovskiy in Kulikovskiy et al. 2012

Achnanthes kryophila subsp. distincta Skvortzov ex Gololobov & Kulikovskiy in Kulikovskiy et al. 2012

Achnanthes kryophila var. sinica Skvortzov ex Gololobov & Kulikovskiy in Kulikovskiy et al. 2012

Achnanthes lanceolata var. argunensis Skvortzov ex Gololobov & Kulikovskiy in Kulikovskiy et al. 2012

Achnanthes lanceolata var. elliptica f. asiatica Skvortzov ex Gololobov & Kulikovskiy in Kulikovskiy et al. 2012

Achnanthes linearis var. szechwanica Skvortzov ex Gololobov & Kulikovskiy in Kulikovskiy et al. 2012

Achnanthes minutissima var. bistriata Skvortzov ex Gololobov & Kulikovskiy in Kulikovskiy et al. 2012

*Achnanthes maolanensis* P.Yu, Kociolek, & Q-M.You in Q-M.You et al. 2019b

Achnanthes montana var. sinica Skvortzov ex Gololobov & Kulikovskiy in Kulikovskiy et al. 2012

*Achnanthes pseudoexigua* Skvortzov ex Gololobov & Kulikovskiy in Kulikovskiy et al. 2012

Achnanthes pseudoexigua var. unilateralis Skvortzov ex Gololobov & Kulikovskiy in Kulikovskiy et al. 2012

*Achnanthes radiata* Skvortzov ex Gololobov & Kulikovskiy in Kulikovskiy et al. 2012

*Achnanthes rarissima* Skvortzov ex Gololobov & Kulikovskiy in Kulikovskiy et al. 2012

*Achnanthes striatella* Skvortzov ex Gololobov & Kulikovskiy in Kulikovskiy et al. 2012

*Achnanthidium epilithica* P.Yu, Q-M.You & Q-X Wang in Yu et al. 2019c

*Achnanthidium guizhouensis* P.Yu, You & Kociolek in You et al. 2019a

*Achnanthidium jiuzhaienis* P.Yu, Q-M.You & Q-X Wang in Yu et al. 2019c

*Achnanthidium lacustre* P.Yu, Q-M. You et Kociolek in Yu et al. 2019b

*Achnanthidium limosua* P.Yu, Q-M. You & Q-X Wang in Yu et al. 2019c

*Achnanthidium longissimum* P.Yu, Q-M.You & Kociolek in Yu et al. 2019a

*Achnanthidium mediolanceolatum* P.Yu, Q-M.You & Kociolek in You et al. 2019a

*Achnanthidium parvulum* You, Q-M.You & Kociolek in You et al. 2019a

*Achnanthidium sinense* B.Liu & S.Blanco in B. Liu et al. 2016

*Achnanthidium sublanceolatum* P.Yu, Q-M.You et Kociolek in Yu et al. 2019b

*Achnanthidium subtilissimum* P.Yu, Q-M.You & Q-X Wang in Yu et al. 2019c

*Achnanthidium taipingensis* P.Yu, Q-M.You et Kociolek in Yu et al. 2019b

*Adlafia sinensis* B.Liu & D.M.Williams in B.Liu et al. 2017

*Amphora chu-yin-changii* Skvortzov ex Gololobov & Kulikovskiy in Kulikovskiy et al. 2012

*Amphora dalaica* Skvortzov ex Gololobov & Kulikovskiy in Kulikovskiy et al. 2012

Amphora dalaica var. bistriata Skvortzov ex Gololobov & Kulikovskiy in Kulikovskiy et al. 2012

Amphora dalaica var. latostriata Skvortzov ex Gololobov & Kulikovskiy in Kulikovskiy et al. 2012

Amphora dalaica var. oculata Skvortzov ex Gololobov & Kulikovskiy in Kulikovskiy et al. 2012

Amphora delicatissima var. dalaica Skvortzov ex Gololobov & Kulikovskiy in Kulikovskiy et al. 2012

Amphora delicatissima var. pekinensis Skvortzov ex Gololobov & Kulikovskiy in Kulikovskiy et al. 2012

*Amphora epithemiformis* Skvortzov ex Gololobov & Kulikovskiy in Kulikovskiy et al. 2012

*Amphora jao* Skvortzov ex Gololobov & Kulikovskiy in Kulikovskiy et al. 2012

*Amphora liouiana* Skvortzov ex Gololobov & Kulikovskiy in Kulikovskiy et al. 2012

*Amphora meyeri* Skvortzov ex Gololobov & Kulikovskiy in Kulikovskiy et al. 2012

Amphora normanii var. alkalina Skvortzov ex Gololobov & Kulikovskiy in Kulikovskiy et al. 2012

Amphora normanii var. curta Skvortzov ex Gololobov & Kulikovskiy in Kulikovskiy et al. 2012

Amphora normanii var. curta f. mongolica Skvortzov ex Gololobov & Kulikovskiy in Kulikovskiy et al. 2012

Amphora normanii var. interrupta Skvortzov ex Gololobov & Kulikovskiy in Kulikovskiy et al. 2012

Amphora normanii var. pekinensis Skvortzov ex Gololobov & Kulikovskiy in Kulikovskiy et al. 2012

Amphora normanii var. poyangi Skvortzov ex Gololobov & Kulikovskiy in Kulikovskiy et al. 2012

Amphora perpusilla var. mongolica Skvortzov ex Gololobov & Kulikovskiy in Kulikovskiy et al. 2012

Amphora perpusilla var. pekinensis Skvortzov ex Gololobov & Kulikovskiy in Kulikovskiy et al. 2012

Amphora perpusilla var. subelliptica Skvortzov ex Gololobov & Kulikovskiy in Kulikovskiy et al. 2012

*Amphora subsalina* Skvortzov ex Gololobov & Kulikovskiy in Kulikovskiy et al. 2012

*Amphora wangchanii* Skvortzov ex Gololobov & Kulikovskiy in Kulikovskiy et al. 2012

*Amphora wang-wei* Skvortzov ex Gololobov & Kulikovskiy in Kulikovskiy et al. 2012

*Aneumastus yamdrokensis* Q.Liu & S.L. Xie in Q.Liu et al. 2018

Caloneis clevei var. parallela Skvortzov ex Gololobov & Kulikovskiy in Kulikovskiy et al. 2012

*Clipeoparvus tibeticus* Q.Liu, Kociolek & Xie in Q.Liu et al. 2019b

Cocconeis pediculus var. cruciata Skvortzov ex Gololobov & Kulikovskiy in Kulikovskiy et al. 2012

Cocconeis pediculus var. emarginata Skvortzov ex Gololobov & Kulikovskiy in Kulikovskiy et al. 2012

Coscinodiscus rothii var. sibirica Skvortzov ex Gololobov & Kulikovskiy in Kulikovskiy et al. 2012

Coscinodiscus sinicus var. sinica Skvortzov ex Gololobov & Kulikovskiy in Kulikovskiy et al. 2012

*Cyclotella changhai* J.-X. Xu & J.P. Kociolek in Xu et al. 2017

Cyclotella glomerata var. argunensis Skvortzov ex Gololobov & Kulikovskiy in Kulikovskiy et al. 2012

Cyclotella kutzingiana subsp. densestriata Skvortzov ex Gololobov & Kulikovskiy in Kulikovskiy et al. 2012

Cyclotella kutzingiana var. dalaica Skvortzov ex Gololobov & Kulikovskiy in Kulikovskiy et al. 2012

Cyclotella meneghiniana var. pumila fo. sibirica Skvortzov ex Gololobov & Kulikovskiy in Kulikovskiy et al. 2012

Cymatopleura elliptica var. sinica Skvortzov ex Gololobov & Kulikovskiy in Kulikovskiy et al. 2012

*Cymatopleura aquastudia* Kociolek & You in You et al. 2017b

*Cymatopleura xinjiangiana* You & Kociolek in You et al. 2017b

*Cymbella chow-yi-liangii* Skvortzov ex Gololobov & Kulikovskiy in Kulikovskiy et al. 2012

Cymbella cistuloides var. angulata Skvortzov ex Gololobov & Kulikovskiy in Kulikovskiy et al. 2012

Cymbella cistuloides var. angulata f. corni-caprae Skvortzov ex Gololobov & Kulikovskiy in Kulikovskiy et al. 2012

Cymbella cistuloides var. angulata f. minor Skvortzov ex Gololobov & Kulikovskiy in Kulikovskiy et al. 2012

Cymbella cistuloides var. bilateralis Skvortzov ex Gololobov & Kulikovskiy in Kulikovskiy et al. 2012

Cymbella cistuloides var. truncata Skvortzov ex Gololobov & Kulikovskiy in Kulikovskiy et al. 2012

Cymbella cistuloides var. undulata Skvortzov ex Gololobov & Kulikovskiy in Kulikovskiy et al. 2012

*Cymbella dalaica* Skvortzov ex Gololobov & Kulikovskiy in Kulikovskiy et al. 2012

Cymbella gracilis var. sinica Skvortzov ex Gololobov & Kulikovskiy in Kulikovskiy et al. 2012

*Cymbella distalebiseriata* B.Liu & D.M.Williams in B.Liu et al. 2018d

*Cymbella fuxianensis* Y.Li & Gong in Gong and Li 2011

*Cymbella hechiensis* Y.Li & W.Zhang in Zhang et al. 2019

*Cymbella heihainensis* Y.Li & Gong in Hu et al. 2013

*Cymbella hubeiensis* Y.Li Y. in Gong et al. 2013

*Cymbella jianghanensis* Y.Li in Gong et al. 2013 (nomen nudum)

*Cymbella khokhensis* Metzeltin, Lange-Bertalot & Li in Metzeltin et al. 2009

*Cymbella liyangensis* Zhang, Jüttner & E.J. Cox, 2018

Cymbella moelleriana var. argunica Skvortzov ex Gololobov & Kulikovskiy in Kulikovskiy et al. 2012

*Cymbella pekinensis* Skvortzov ex Gololobov & Kulikovskiy in Kulikovskiy et al. 2012

*Cymbella paenetruncata* Y.Li & Z.Gong in Gong et al. 2013

*Cymbella pamirensis* Z.Zhang & Rioual in Z.Zhang et al. 2017

*Cymbella pseudotumida* Skvortzov ex Gololobov & Kulikovskiy in Kulikovskiy et al. 2012

Cymbella pseudotumida var. pseduoborealis Skvortzov ex Gololobov & Kulikovskiy in Kulikovskiy et al. 2012

Cymbella rupicola var. sinica Skvortzov ex Gololobov & Kulikovskiy in Kulikovskiy et al. 2012

Cymbella semicircularis var. dalaica Skvortzov ex Gololobov & Kulikovskiy in Kulikovskiy et al. 2012

Cymbella sinica var. rostrata Skvortzov ex Gololobov & Kulikovskiy in Kulikovskiy et al. 2012

Cymbella sinuata var. argunensis Skvortzov ex Gololobov & Kulikovskiy in Kulikovskiy et al. 2012

Cymbella tumida var. convergentistriata Skvortzov ex Gololobov & Kulikovskiy in Kulikovskiy et al. 2012

*Cymbella tungtingiana* Skvortzov ex Gololobov & Kulikovskiy in Kulikovskiy et al. 2012

*Cymbella pulchra* Y.Li & Lange-Bertalot in Gong et al. 2013

*Cymbella shii* Y.Li in Gong et al. 2013

*Cymbella shudunensis* YLi & Metzeltin in Hu et al. 2013

*Cymbella sinensis* Metzeltin & Krammer in Krammer 2002

*Cymbella xingyunnensis* Y.Li & Gong in Hu et al. 2013

Cymbella yabe var. punctata Y.Li & Shi in Li et al. 2003

*Cymbella yangtzensis* Y.Li & D.Metzeltin in Gong et al. 2013

*Cymbopleura pseudokuelbsii* Shi 2013

*Delicata changqingensis* W.Zhang, S.Q.Yang & S. Blanco in Zhang et al. 2019

*Delicata sinensis* Krammer and Metzeltin 2003

*Delicata williamsii* B.Liu & S. Blanco in Bing Liu et al. 2018b

*Diatoma kalakulensis* Peng, Rioual & D.M. Williams, 2017

*Diatoma rupestris* Y.Liu & Wang in Y.Liu et al. 2010

*Diploneis barbatula* Skvortzov ex Gololobov & Kulikovskiy in Kulikovskiy et al. 2012

Diploneis parma var. sinoborealis Skvortzov ex Gololobov & Kulikovskiy in Kulikovskiy et al. 2012

Diploneis pseudoovalis var. tiensinensis Skvortzov ex Gololobov & Kulikovskiy in Kulikovskiy et al. 2012

Diploneis smithii var. denseareolata Skvortzov ex Gololobov & Kulikovskiy in Kulikovskiy et al. 2012

***Edtheriotia*** Kociolek, Q.You, Stepanek, R.L.Lowe & Q-X.Wang, 2016a

*Edtheriotia guizhoiana* Kociolek, You, Stepanek, R.L.Lowe & Q-X.Wang 2016a

*Entomoneis triundulata* B.Liu & D.M.Williams in B.Liu et al. 2018c

*Epithemia arguiformis* Q-M.You & Q-X.Wang, 2009

*Eucocconeis lichunhaii* Y.Li in Y.Li and Gong 2013

Eunotia arcus var. undulata Skvortzov ex Gololobov & Kulikovskiy in Kulikovskiy et al. 2012

Eunotia bigibba var. subcapitata Skvortzov ex Gololobov & Kulikovskiy in Kulikovskiy et al. 2012

Eunotia diodon var. fukinensis Skvortzov ex Gololobov & Kulikovskiy in Kulikovskiy et al. 2012

*Eunotia filiformis* Luo et al. 2019

Eunotia monodon var. amoyensis Skvortzov ex Gololobov & Kulikovskiy in Kulikovskiy et al. 2012

Eunotia pectinalis var. amoyensis Skvortzov ex Gololobov & Kulikovskiy in Kulikovskiy et al. 2012

*Eunotia mugecuo* Luo et al. 2019

Eunotia pectinalis var. bigibba Skvortzov ex Gololobov & Kulikovskiy in Kulikovskiy et al. 2012

Eunotia pectinalis var. curta Skvortzov ex Gololobov & Kulikovskiy in Kulikovskiy et al. 2012

Eunotia pectinalis var. sinica Skvortzov ex Gololobov & Kulikovskiy in Kulikovskiy et al. 2012

Eunotia praerupta var. sinica Skvortzov ex Gololobov & Kulikovskiy in Kulikovskiy et al. 2012

*Eunotia shantungensis* Skvortzov ex Gololobov & Kulikovskiy in Kulikovskiy et al. 2012

Eunotia shantungensis var. linealata Skvortzov ex Gololobov & Kulikovskiy in Kulikovskiy et al. 2012

*Eunotia sudeticiformis* Kociolek et al. 2016b

Eunotia tauntoniensis var. amoyensis Skvortzov ex Gololobov & Kulikovskiy in Kulikovskiy et al. 2012

Eunotia tridentula var. sinica Skvortzov ex Gololobov & Kulikovskiy in Kulikovskiy et al. 2012

Eunotia valida var. densistriata Skvortzov ex Gololobov & Kulikovskiy in Kulikovskiy et al. 2012

Eunotia valida var. sinica Skvortzov ex Gololobov & Kulikovskiy in Kulikovskiy et al. 2012

Fragilaria crenophila var. sinensis Rioual in Rioual et al. 2017a

*Geissleria jianghanensis* Y.Li inY. Li et al. 2005

*Germainiella guizhouiana* Kociolek et al. 2019b

*Germainiella maolaniana* Kociolek et al. 2019b

*Germainiella sinica* Kociolek et al. 2019b

*Gomphocymbella asymmetrica* Shi & Y.Li in Shi et al. 2003

*Gomphocymbella laxistriata* Shi & Y.Li in Shi et al. 2003

*Gomphoneis distorta* Q-M.You & Kociolek in Q-M. You et al. 2013

*Gomphoneis pseudosubtiloides* Q-M.You & Kociolek in Q-M. You et al. 2013

*Gomphoneis qii* Q-M.You & Kociolek in Q-M. You et al. 2013

*Gomphoneis rostratoides* Q-M.You & Kociolek in Q-M. You et al. 2013

*Gomphoneis stoermeri* Q-M.You & Kociolek in Q-M. You et al. 2013

*Gomphoneis subtiloides* Q-M.You & Kociolek in Q-M. You et al. 2013

*Gomphoneis xinjiangiana* Q-M.You & Kociolek in Q-M. You et al. 2013

Gomphonema acuminatum var. obtusum Y.Fan & Bao in Fan et al. 2004

*Gomphonema argunensis* Skvortzov ex Gololobov & Kulikovskiy in Kulikovskiy et al. 2012

*Gomphonema asiaticum* Y.Liu & Kociolek in Y.Liu et al. 2013

Gomphonema augur var. poyangiana Skvortzov ex Gololobov & Kulikovskiy in Kulikovskiy et al. 2012

Gomphonema clevei var. oryzae Skvortzov ex Gololobov & Kulikovskiy in Kulikovskiy et al. 2012

Gomphonema constrictum var. tumidum Skvortzov ex Gololobov & Kulikovskiy in Kulikovskiy et al. 2012

*Gomphonema gordejevi* Skvortzov ex Gololobov & Kulikovskiy in Kulikovskiy et al. 2012

Gomphonema heideni var. mingiana Skvortzov ex Gololobov & Kulikovskiy in Kulikovskiy et al. 2012

Gomphonema intricatum var. curvatum Skvortzov ex Gololobov & Kulikovskiy in Kulikovskiy et al. 2012

*Gomphonema jao* Skvortzov ex Gololobov & Kulikovskiy in Kulikovskiy et al. 2012

Gomphonema kaznakowi var. mingiana Skvortzov ex Gololobov & Kulikovskiy in Kulikovskiy et al. 2012

*Gomphonema bicepiformis* Zhang & Kociolek,2018b

*Gomphonema bigutianchnensis* Y.Li in Liao and Y.Li 2018

*Gomphonema chinense* Y.Liu & Kociolek in Y.Liu et al. 2013

Gomphonema constrictum var. ellipticum Z.X.Shi & J.Y.Chen in Shi 2004

*Gomphonema dichotiforme* Z.X.Shi, 2004

*Gomphonema genestoermeri* Y.Liu & Kociolek in Y.Liu et al. 2013

*Gomphonema heilongtanensis* Y.Li, Kociolek & D.Metzeltin, 2010

Gomphonema instabilis var. rhombicum S.Q.Xie & Z.H.Shi in Shi 2004

*Gomphonema intricatoides* Q-M.You & Kociolek, 2015

Gomphonema intricatum var. mirum Z.X.Shi & H.Z.Zhu, 2004

*Gomphonema jianghanense* Y.Li, Z.J.Gong, P.Xie & J.Shen, 2007

Gomphonema kaznakowi var. cruciatum Z.X.Shi & Y.Li in Shi 2014

Gomphonema lanceolatum var. amuricum Skvortzov ex Gololobov & Kulikovskiy in Kulikovskiy et al. 2012

Gomphonema lanceolatum var. curtum Skvortzov ex Gololobov & Kulikovskiy in Kulikovskiy et al. 2012

Gomphonema longiceps var. rupestris Skvortzov ex Gololobov & Kulikovskiy in Kulikovskiy et al. 2012

*Gomphonema mediocris* Skvortzov ex Gololobov & Kulikovskiy in Kulikovskiy et al. 2012

Gomphonema mediocris var. capitatum Skvortzov ex Gololobov & Kulikovskiy in Kulikovskiy et al. 2012

*Gomphonema mereschkowskyii* Skvortzov ex Gololobov & Kulikovskiy in Kulikovskiy et al. 2012

Gomphonema mereschkowskyii subsp. lancetulum Skvortzov ex Gololobov & Kulikovskiy in Kulikovskiy et al. 2012

Gomphonema olivaceum var. argunensis Skvortzov ex Gololobov & Kulikovskiy in Kulikovskiy et al. 2012

*Gomphonema metzeltinii* Q-M.You & Kociolek, 2015

*Gomphonema microlanceolatum* Q-M.You & Kociolek, 2015

Gomphonema montanum var. multipunctatum Z.X.Shi & H.Z.Zhu in Shi 2004

Gomphonema olivaceum var. brevistriatum Li & Shi in Y.Li et al. 2003

Gomphonema olivaceum var. brevistriatum Y.Li & Shi in Li et al. 2003

Gomphonema olivaceum var. densostriatum Z.X.Shi & H.Z.Zhu, 2004

Gomphonema olivaceum var. punctatum Z.X.Shi & N. Li in Shi 2004

*Gomphonema pygmaeoides* Q-M.You & Kociolek, 2015

*Gomphonema rexlowei* Y.Liu & Kociolek in Y.Liu et al. 2013

*Gomphonema shanghaiensis* Zhang & Kociolek, 2016b

Gomphonema shangtungensis var. rostratum Skvortzov ex Gololobov & Kulikovskiy in Kulikovskiy et al. 2012

*Gomphonema sichuanensis* Y.Li & Kociolek in Li et al. 2010b

Gomphonema staurophorum var. oblongum Li & Shi in Li et al. 2003

Gomphonema subclavatum var. elongatum Z.X.Shi, 2014

*Gomphonema subinsigniforme* L.Ge, Y.Liu & Kociolek, 2014

Gomphonema turris var. elongatum Z.X. Shi & H.Z. Zhu in Shi 2004

Gomphonema turris var. latum Y.Fan & Q-X.Wang in Fan et al. 2004

*Gomphonema wangii* Skvortzov ex Gololobov & Kulikovskiy in Kulikovskiy et al. 2012

*Gomphonema williamsii* Kociolek & Y.Liu in Y.Liu et al. 2013

*Gomphonema witkowskii* Kociolek & Y. Liu in Y.Liu et al. 2013

*Gomphonema wuyiensis* W.Zhang & Kociolek in Zhang et al. 2018

*Gomphonema xiantaoicum* Z.X.Shi & N.Li in Z.X.Shi 2014

*Gomphonema xiantaoicum* Z.X.Shi & N.Li in Z.X.Shi 2014

*Gomphonema xinjiangianum* Q-M.You & Kociolek, 2015

*Gomphonema yangtzensis* Y.Li in Y.Li et al. 2006

*Gomphonema yaominae* Y.Li, in Gong and Li 2012

*
Gomphosinica* Kociolek et al. 2015

*Gomphosinica capitata* Kociolek, Q-M.You & Q-X. Wang in Kociolek et al. 2015

*Gomphosinica lugunsis* Y.Liu et al. in Cheng et al. 2018

*Gomphosinica robusta* Kociolek, Q-M.You & Q-X. Wang in Kociolek et al. 2015

*Gomphosinica selincuoensis* Z.Zhang, Q-M.You & Kociolek in Yang et al. 2019

*Gomphosinica simsiae* Kociolek, Q-M.You & Q-X.Wang in Kociolek et al. 2015

*Gomphosinica subtilis* Kociolek, Q-M.You & Q-X.Wang in Kociolek et al. 2015

Gyrosigma peisonis var. major Peng, Rioual & Sterrenburg, 2016

*Halamphora daochengensis* Zhang, Jüttner & Levkov in Zhang et al. 2019

*Halamphora hezhangii* Q-M.You & Kociolek in Q-M. You et al. 2015c

*Halamphora subfontinalis* Q-M.You & Kociolek in Q-M. You et al. 2015c

*Hannaea tibetiana* Q.Liu, Glushchenko, Kulikovskiy & Kociolek in Q.Liu et al. 2019a

*Hantzschia lineolata* Skvortzov ex Gololobov & Kulikovskiy in Kulikovskiy et al. 2012

Hantzschia virgata var. dalaica Skvortzov ex Gololobov & Kulikovskiy in Kulikovskiy et al. 2012

*Hippodonta qinghainensis* Peng & Rioual, 2014

*Humidophila cavernaphila* Lowe, Kociolek & Q-M.You in Lowe et al. 2017

*Humidophila minuta* Lowe, Kociolek & Q-M.You in Lowe et al. 2017

*Humidophila panduriformis* Lowe, Kociolek & Q-M.You in Lowe et al. 2017

*Humidophila potapovae* Lowe, Kociolek & Q-M.You in Lowe et al. 2017

*Humidophila undulocontenta* Lowe, Kociolek & Q-M.You in Lowe et al. 2017

*Kolbesia sichuanenis* P. Yu, Q-M. You & Q-X. Wang in Yu et al. 2019a

*Lindavia khinganensis* Rioual in Rioual et al. 2017

*Luticola hunanensis* B.Liu & D.M.Williams in B.Liu et al. 2017a

*Luticola wulingensis* B.Liu & S.Blanco in B.Liu et al. 2017a

*Melosira asiatica* Skvortzov ex Gololobov & Kulikovskiy in Kulikovskiy et al. 2012

*Muelleria pseudogibbula* Q.Liu & Q-X.Wang in Liu et al. 2018

*Navicula craticuloides* Li & Metzeltin in Gong et al. 2015

*Navicula gongii* Metzeltin & Li in Gong et al. 2015

*Navicula ignorata* Skvortzov ex Gololobov & Kulikovskiy in Kulikovskiy et al. 2012

Navicula salinarum f. gracilis Skvortzov ex Gololobov & Kulikovskiy in Kulikovskiy et al. 2012

Navicula subocculata var. parallelistriata Skvortzov ex Gololobov & Kulikovskiy in Kulikovskiy et al. 2012

*Navicula wangii* Skvortzov ex Gololobov & Kulikovskiy in Kulikovskiy et al. 2012

Navicula wangii f. constricta Skvortzov ex Gololobov & Kulikovskiy in Kulikovskiy et al. 2012

Navicula wangii var. obtusa Skvortzov ex Gololobov & Kulikovskiy in Kulikovskiy et al. 2012

Navicula wangii var. subcapitata Skvortzov ex Gololobov & Kulikovskiy in Kulikovskiy et al. 2012

Navicula seposita var. major Chen & Zhu in Zhu and Chen 2000

Navicula subtilissima var. paucistriata Chen & Zhu in Zhu and Chen 2000

*Navicula yunnanensis* Li & Metzeltin in Gong et al. 2015

*Neidiomorpha sichuaniana* Q.Liu, Q-X.Wang & Kociolek, in Q.Liu et al. 2014a

*Neidium angustatum* Q.Liu, Q-X.Wang & Kociolek in Q.Liu et al. 2017

*Neidium apiculatoides* Q.Liu, Q-X.Wang & Kociolek in Q.Liu et al. 2017

*Neidium avenaceum* Q.Liu, Q-X.Wang & Kociolek in Q.Liu et al. 2017

*Neidium bacillum* Q.Liu, Q-X.Wang & Kociolek in Q.Liu et al. 2017

*Neidium chenii* Y.Liu & Kociolek in Y.Liu et al. 2014a

*Neidium convexum* Q.Liu, Q-X.Wang & Kociolek in Q.Liu et al. 2017

*Neidium dicephalum* Q.Liu, Q.X.Wang & Kociolek in Liu et al. 2017

Neidium hitchcockii var. obliquestriatum f. densestriatum Skvortzov ex Gololobov & Kulikovskiy in Kulikovskiy et al. 2012

*Neidium lacusflorum* Q.Liu, Q-X.Wang & Kociolek in Q.Liu et al. 2017

*Neidium ligulatum* Q.Liu, Q-X.Wang & Kociolek in Q.Liu et al. 2017

*Neidium limuae* Y.Liu & Kociolek, 2014 in Y.Liu et al. 2014a

*Neidium qia* Q.Liu, Q-X.Wang & Kociolek in Q.Liu et al. 2017

*Neidium rostellatum* Q.Liu, Q-X.Wang & Kociolek in Q.Liu et al. 2017

*Neidium rostratum* Q.Liu, Q-X.Wang & Kociolek in Q.Liu et al. 2017

*Neidium suboblongum* Q.Liu, Q-X.Wang & Kociolek in Q.Liu et al. 2017

*Neidium suoxiyuae** Y.Liu & Kociolek in Y.Liu et al. 2014a

*Neidium tibetianum* Q.Liu, Q-X.Wang & Kociolek in Q.Liu et al. 2017

*Neidium tibeticum** Y.Liu & J.P.Kociolek in Y.Liu et al. 2014a

*Neidium tortum* Q.Liu, Q-X.Wang & Kociolek in Q. Liu et al. 2017

*Neidium triundulatum* Q.Liu, Q-X.Wang & Kociolek in Q.Liu et al. 2017

*Neidium zhui** Y.Liu & J.P.Kociolek in Y.Liu et al. 2014a

*Neidium zoigeaeum* Q.Liu, Q-X.Wang & Kociolek in Q.Liu et al. 2017

Nitzschia acuta var. argunensis Skvortzov ex Gololobov & Kulikovskiy in Kulikovskiy et al. 2012

Nitzschia angustata var. dalaica Skvortzov ex Gololobov & Kulikovskiy in Kulikovskiy et al. 2012

Nitzschia apiculata var. latostriata Skvortzov ex Gololobov & Kulikovskiy in Kulikovskiy et al. 2012

Nitzschia fonticola var. acuta Skvortzov ex Gololobov & Kulikovskiy in Kulikovskiy et al. 2012

Nitzschia gracilis var. minuta Skvortzov ex Gololobov & Kulikovskiy in Kulikovskiy et al. 2012

*Nitzschia hastata* Skvortzov ex Gololobov & Kulikovskiy in Kulikovskiy et al. 2012

Nitzschia hastata var. obtusa Skvortzov ex Gololobov & Kulikovskiy in Kulikovskiy et al. 2012

Nitzschia hastata var. parallelistriata Skvortzov ex Gololobov & Kulikovskiy in Kulikovskiy et al. 2012

Nitzschia intermedia var. sinica Skvortzov ex Gololobov & Kulikovskiy in Kulikovskiy et al. 2012

*Nitzschia kalganica* Skvortzov ex Gololobov & Kulikovskiy in Kulikovskiy et al. 2012

Nitzschia linearis var. robustrior Skvortzov ex Gololobov & Kulikovskiy in Kulikovskiy et al. 2012

Nitzschia obtusa var. minuta Skvortzov ex Gololobov & Kulikovskiy in Kulikovskiy et al. 2012

Nitzschia parvula var. recta Skvortzov ex Gololobov & Kulikovskiy in Kulikovskiy et al. 2012

Nitzschia recta var. lanceolata Skvortzov ex Gololobov & Kulikovskiy in Kulikovskiy et al. 2012

Nitzschia recta var. tenuirostris Skvortzov ex Gololobov & Kulikovskiy in Kulikovskiy et al. 2012

*Nitzschia sheshukowae* Skvortzov ex Gololobov & Kulikovskiy in Kulikovskiy et al. 2012

Nitzschia sinuata var. undulata Skvortzov ex Gololobov & Kulikovskiy in Kulikovskiy et al. 2012

*Nitzschia tryblionella f. obtusiuscula mongolica* Skvortzov ex Gololobov & Kulikovskiy in Kulikovskiy et al. 2012

Nitzschia tryblionella var. tungtingiana Skvortzov ex Gololobov & Kulikovskiy in Kulikovskiy et al. 2012

*Nitzschia tungtingiana* Skvortzov ex Gololobov & Kulikovskiy in Kulikovskiy et al. 2012

*Nitzschia wangtzianii* Skvortzov ex Gololobov & Kulikovskiy in Kulikovskiy et al. 2012

*Nupela major* P.Yu, Q-M.You & Kociolek in Yu et al. 2017

*Oricymba rhynchocephala* Zhang & Kociolek in Zhang et al. 2018c

*Oricymba tianmuensis* Zhang & Li in Zhang et al. 2015

*Oricymba xianjuensis* Zhang & Kociolek in Zhang et al. 2016a

*Pinnularia amurensis* Skvortzov ex Gololobov & Kulikovskiy in Kulikovskiy et al. 2012

Pinnularia appendiculata var. densestriata Skvortzov ex Gololobov & Kulikovskiy in Kulikovskiy et al. 2012

*Pinnularia argunensis* Skvortzov ex Gololobov & Kulikovskiy in Kulikovskiy et al. 2012

Pinnularia balfouriana var. brevicostata Skvortzov ex Gololobov & Kulikovskiy in Kulikovskiy et al. 2012

*Pinnularia aquaedulcis* Y.Liu, Kociolek & Q-X.Wang in Kociolek et al. 2018

Pinnularia borealis var. densestriata Skvortzov ex Gololobov & Kulikovskiy in Kulikovskiy et al. 2012

Pinnularia braunii var. angustata Skvortzov ex Gololobov & Kulikovskiy in Kulikovskiy et al. 2012

Pinnularia braunii var. curta Skvortzov ex Gololobov & Kulikovskiy in Kulikovskiy et al. 2012

Pinnularia ceylonica var. costulata Skvortzov ex Gololobov & Kulikovskiy in Kulikovskiy et al. 2012

Pinnularia ceylanica var. gigantea Skvortzov, 2012 (original description)

*Pinnularia composita* Skvortzov ex Gololobov & Kulikovskiy in Kulikovskiy et al. 2012

Pinnularia composita var. acuta Skvortzov ex Gololobov & Kulikovskiy in Kulikovskiy et al. 2012

Pinnularia composita var. distincta Skvortzov ex Gololobov & Kulikovskiy in Kulikovskiy et al. 2012

Pinnularia composita var. linearis Skvortzov ex Gololobov & Kulikovskiy in Kulikovskiy et al. 2012

Pinnularia dactylus var. convergentissima Skvortzov ex Gololobov & Kulikovskiy in Kulikovskiy et al. 2012

Pinnularia dactylus var. mingiana Skvortzov ex Gololobov & Kulikovskiy in Kulikovskiy et al. 2012

Pinnularia dactylus var. semitropica Skvortzov ex Gololobov & Kulikovskiy in Kulikovskiy et al. 2012

*Pinnularia dalaica* Skvortzov ex Gololobov & Kulikovskiy in Kulikovskiy et al. 2012

*Pinnularia clavata* Y.Liu, Kociolek & Q-X.Wang in Y.Liu et al. 2018a

*Pinnularia crater-lapis* Y.Liu, Kociolek & Q-X.Wang in Y.Liu et al. 2018a

*Pinnularia daerbinsis* Y.Liu, Kociolek & Q-X.Wang in Y.Liu et al. 2018a

*Pinnularia dicephala* Y.Liu, Kociolek & Q-X.Wang in Y.Liu et al. 2018a

*Pinnularia distans* Y.Liu, Kociolek & Q-X.Wang in Y.Liu et al. 2018a

*Pinnularia elliptica* Y.Liu, Kociolek & Q-X.Wang in Y.Liu et al. 2018a, accepted as *Pinnularia palidis* Y.Liu, Kociolek & Q-X.Wang in Kociolek et al. 2018

Pinnularia episcopalis var. mingiana Skvortzov ex Gololobov & Kulikovskiy in Kulikovskiy et al. 2012

Pinnularia gibba var. mingiana Skvortzov ex Gololobov & Kulikovskiy in Kulikovskiy et al. 2012

Pinnularia gibba var. lata Skvortzov ex Gololobov & Kulikovskiy in Kulikovskiy et al. 2012

*Pinnularia gigantea* Skvortzov ex Gololobov & Kulikovskiy in Kulikovskiy et al. 2012

Pinnularia gigantea var. interrupta Skvortzov ex Gololobov & Kulikovskiy in Kulikovskiy et al. 2012

Pinnularia gigantea var. minor Skvortzov ex Gololobov & Kulikovskiy in Kulikovskiy et al. 2012

Pinnularia hartleyana var. amurensis Skvortzov ex Gololobov & Kulikovskiy in Kulikovskiy et al. 2012

*Pinnularia gracile* Y.Liu, Kociolek & Q-X.Wang in Y.Liu et al. 2018a

Pinnularia hemiptera var. longilineata Skvortzov ex Gololobov & Kulikovskiy in Kulikovskiy et al. 2012

Pinnularia interrupta var. tungtingiana Skvortzov ex Gololobov & Kulikovskiy in Kulikovskiy et al. 2012

*Pinnularia jao* Skvortzov ex Gololobov & Kulikovskiy in Kulikovskiy et al. 2012

*Pinnularia kolbei* Skvortzov ex Gololobov & Kulikovskiy in Kulikovskiy et al. 2012

*Pinnularia krasskei* Skvortzov ex Gololobov & Kulikovskiy in Kulikovskiy et al. 2012

Pinnularia krasskei var. latior Skvortzov ex Gololobov & Kulikovskiy in Kulikovskiy et al. 2012

Pinnularia lacushankae var. convergenda Skvortzov ex Gololobov & Kulikovskiy in Kulikovskiy et al. 2012

Pinnularia lata var. amurensis Skvortzov ex Gololobov & Kulikovskiy in Kulikovskiy et al. 2012

Pinnularia lata var. linearis Skvortzov ex Gololobov & Kulikovskiy in Kulikovskiy et al. 2012

Pinnularia legumen var. sinica Skvortzov ex Gololobov & Kulikovskiy in Kulikovskiy et al. 2012

*Pinnularia liouniata* Skvortzov ex Gololobov & Kulikovskiy in Kulikovskiy et al. 2012

Pinnularia major var. sinica Skvortzov ex Gololobov & Kulikovskiy in Kulikovskiy et al. 2012

*Pinnularia meisteriana* Skvortzov ex Gololobov & Kulikovskiy in Kulikovskiy et al. 2012

*Pinnularia montium* Y.Liu, Kociolek & Q-X.Wang in Kociolek et al. 2018a

*Pinnularia palidis* Y.Liu, Kociolek & Q-X.Wang in Kociolek et al. 2018a

*Pinnularia paliobducta* Y.Liu, Kociolek & Q-X.Wang in Kociolek et al. 2018a

*Pinnularia paludosa* Y.Liu & Q-X.Wang, 2010 in Y.Liu et al. 2010b

*Pinnularia paludosa* Y.Liu & Q-XWang, 2010 in Y.Liu et al. 2010b

*Pinnularia parallela* Y.Liu, Kociolek & Q-X.Wang in Y.Liu et al. 2018a, accepted as *Pinnularia shii* Y.Liu, Kociolek & Q-X.Wang in Kociolek et al. 2018a

*Pinnularia parvulum* Y.Liu, Kociolek & Q-X.Wang in Y.Liu et al. 2018a

Pinnularia platycephala var. nipponica Skvortzov ex Gololobov & Kulikovskiy in Kulikovskiy et al. 2012

Pinnularia polyonca var. nipponica f. australis Skvortzov ex Gololobov & Kulikovskiy in Kulikovskiy et al. 2012

*Pinnularia pseudosinistra* Y.Liu, Kociolek & Q-X.Wang in Y.Liu et al. 2018a

*Pinnularia qii* Y.Liu, Kociolek & Q.X.Wang in Y.Liu et al. 2018a

*Pinnularia rectangularis* Y.Liu, Kociolek & Q-X.Wang in Y.Liu et al. 2018a

*Pinnularia shii* Y.Liu, Kociolek & Q.X.Wang in Kociolek et al. 2018a

*Pinnularia sinicorum* Skvortzov ex Gololobov & Kulikovskiy in Kulikovskiy et al. 2012

Pinnularia stauroptera var. mingiana Skvortzov ex Gololobov & Kulikovskiy in Kulikovskiy et al. 2012

Pinnularia stauroptera var. rostrata Skvortzov ex Gololobov & Kulikovskiy in Kulikovskiy et al. 2012

Pinnularia streptoraphe var. argunensis Skvortzov ex Gololobov & Kulikovskiy in Kulikovskiy et al. 2012

Pinnularia streptoraphe var. tumida Skvortzov ex Gololobov & Kulikovskiy in Kulikovskiy et al. 2012

Pinnularia subcapitata var. mingiana Skvortzov ex Gololobov & Kulikovskiy in Kulikovskiy et al. 2012

*Pinnularia subaldenii* Q-X.Wang & Y.Liu, 2010 in Y. Liu et al. 2010b

*Pinnularia subbrebissonii* Y.Liu, Kociolek & Q.X.Wang in Y.Liu et al. 2018a

*Pinnularia submicrostauron* Y.Liu, Kociolek & Q.X.Wang in Y.Liu et al. 2018a, accepted as *Pinnularia aquaedulcis* Y.Liu, Kociolek & Q.X.Wang in Kociolek et al. 2018

*Pinnularia subnotabilis* Y.Liu, Kociolek & Q.X.Wang in Y.Liu et al. 2018a

*Pinnularia subobscura* Y.Liu, Kociolek & Q.X.Wang in Y.Liu et al. 2018a

Pinnularia subsalaris var. sinica Skvortzov ex Gololobov & Kulikovskiy in Kulikovskiy et al. 2012

Pinnularia tabellaria var. sinica Skvortzov ex Gololobov & Kulikovskiy in Kulikovskiy et al. 2012

*Pinnularia tschangbaischanica* Skvortzov ex Gololobov & Kulikovskiy in Kulikovskiy et al. 2012

*Pinnularia subsinistra* Y.Liu, Kociolek & Q.X.Wang in Y.Liu et al. 2018a, accepted as *Pinnularia paliobducta* Y.Liu, Kociolek & Q.X.Wang in Kociolek et al. 2018

*Pinnularia wuyiensis* Zhang et al. 2016

*Pinnularia xianhensis* Y.Liu, Kociolek & Q.X.Wang in Y.Liu et al. 2018a

*Pinnularia zebra* Y.Liu, Kociolek & Q.X.Wang in Y.Liu et al. 2018a

*Placoneis sinensis* Y.Li & D.Metzeltin in Gong et al. 2013

*Platessa guangzhouae* Y.Liu & Kociolek in Y.Liu et al. 2014b

*Pliocaenicus changbaiense* Stachura-Suchoples and Jahn 2009

*Prestauroneis lowei* Liu, Wang & Kociolek, 2014

*Prestauroneis nenwai* Liu, Wang & Kociolek, 2014

*Psammothidium hainanii* Kociolek & Y.Liu, in Liu et al. 2014

Rhopalodia gibba var. dalaica Skvortzov ex Gololobov & Kulikovskiy in Kulikovskiy et al. 2012

*Rhopalodia pseudogibba* Skvortzov ex Gololobov & Kulikovskiy in Kulikovskiy et al. 2012

Rhopalodia pseudogibba var. arcuata Skvortzov ex Gololobov & Kulikovskiy in Kulikovskiy et al. 2012

Rhopalodia pseudogibba var. pseudoventricosa Skvortzov ex Gololobov & Kulikovskiy in Kulikovskiy et al. 2012

Stauroneis javanica var. truncata Skvortzov ex Gololobov & Kulikovskiy in Kulikovskiy et al. 2012

*Stenopterobia recta* Skvortzov ex Gololobov & Kulikovskiy in Kulikovskiy et al. 2012

*Stephanodiscus argunensis* Skvortzov ex Gololobov & Kulikovskiy in Kulikovskiy et al. 2012

Stephanodiscus argunensis var. simple Skvortzov ex Gololobov & Kulikovskiy in Kulikovskiy et al. 2012

*Stephanodiscus hustedtii* Skvortzov ex Gololobov & Kulikovskiy in Kulikovskiy et al. 2012

*Sellaphora constrictum* Kociolek & Q-M.You in Q-M.You et al. 2017a

*Sellaphora sinensis* Y.Li & Metzeltin in Y.Li et al. 2013

*Sellaphora yunnanensis* Y.Li & Metzeltin in Y.Li et al. 2013

*Sellaphora yunnanensis* Y.Li in Y.Li et al. 2010c

*Sellphora fuxianensis* Y.Li in Y.Li et al. 2010a

***Sichuania*** Y.Li, H. Lange-Bertalot & D. Metzeltin, 2009 accepted as *Sichuaniella* Li Yanling, Lange-Bertalot & Metzeltin, 2013

*Sichuania lacustris* Y.Li, H.Lange-Bertalot & D.Metzelin, 2009

***Sichuaniella*** Y.Li, Lange-Bertalot & Metzeltin, 2013

*Sichuaniella lacustris* (Y.Li, Lange-Bertalot & Metzeltin) Y.Li, Lange-Bertalot & Metzeltin, 2013

*Simonsenia maolaniana* Q-M.You & Kociolek, in Q-M.You et al. 2016

***Sinoperonia*** Kociolek, Y.Liu, Glushchenko & Kulikovskiy in Y.Liu et al. 2018b

*Sinoperonia polyraphiamorpha* Kociolek, Y.Liu, Glushchenko & Kulikovskiy in Y.Liu et al. 2018b

*Stauroneis lacusvulcani* P.Rioual, 2013

*Staurosira longwanensis* P.Rioual, Morales & Ector, 2014

Surirella angustata var. apiculata Skvortzov ex Gololobov & Kulikovskiy in Kulikovskiy et al. 2012

Surirella tientsinensis var. striolata Skvortzov ex Gololobov & Kulikovskiy in Kulikovskiy et al. 2012

*Surirella wulingensis* B.Liu & Ector in B.Liu et al. 2019c

*Synedra amoyensis* Skvortzov ex Gololobov & Kulikovskiy in Kulikovskiy et al. 2012

*Synedra arguinensis* Skvortzov ex Gololobov & Kulikovskiy in Kulikovskiy et al. 2012

*Synedra chungii* Skvortzov ex Gololobov & Kulikovskiy in Kulikovskiy et al. 2012

*Synedra sinica* Skvortzov ex Gololobov & Kulikovskiy in Kulikovskiy et al. 2012

Synedra sinica var. recta Skvortzov ex Gololobov & Kulikovskiy in Kulikovskiy et al. 2012

Synedra tenera var. subtenera Skvortzov ex Gololobov & Kulikovskiy in Kulikovskiy et al. 2012

Synedra ulna var. repanda Wang & You in You et al. 2008

*Tabularia sinensis* Cao et al. 2018

***Tibetiella*** Y.Li, D.M.Williams & D.Metzeltin, 2010d

*Tibetiella pulchra* Y.Li, D.M.Williams & Metzeltin, 2010d

*Ulnaria dongtingensis* B.Liu in B.Liu et al. 2019b

*Ulnaria gaowangjiensis* B.Liu & D.M.Williams in B.Liu et al. 2017

*Ulnaria jinbianensis* S.Blanco & B.Liu in B.Liu et al. 2019b

*Ulnaria oxybiseriata* D.M.Williams & B.Liu In B.Liu et al. 2019b

*Ulnaria rhombus* D.M.Williams in B.Liu et al. 2019a

*Ulnaria sinensis* B.Liu & D.M.Williams in B.Liu et al. 2017

*Ulnaria ulnabiseriata* D.M.Williams & B.Liu in B.Liu et al. 2017

*Ulnaria wulingensis* B.Liu in B.Liu et al. 2019a

*Urosolenia subtenuis* Kociolek & Y.Liu in Y.Liu et al. 2016

*Urosolenia truncata* Y.Liu & Kociolek in Y.Liu et al. 2016

*Urosolenia yalongii* Kociolek & Y. Liu in Y.Liu et al. 2016
